# Conservation of the conformational dynamics and ligand binding within M49 enzyme family†

**DOI:** 10.1039/c7ra13059g

**Published:** 2018-04-10

**Authors:** Saša Kazazić, Zrinka Karačić, Igor Sabljić, Dejan Agić, Marko Tomin, Marija Abramić, Michal Dadlez, Antonija Tomić, Sanja Tomić

**Affiliations:** Ruđer Bošković Institute, Institute of Biochemistry and Biophysics Polish Academy of Sciences Croatia sasa.kazazic@irb.hr; Ruđer Bošković Institute, Institute of Biochemistry and Biophysics Polish Academy of Sciences Croatia sanja.tomic@irb.hr; Josip Juraj Strossmayer University of Osijek, Faculty of Agriculture Croatia; Institute of Biochemistry and Biophysics Polish Academy of Sciences Poland

## Abstract

The hydrogen deuterium exchange (HDX) mass spectrometry combined with molecular dynamics (MD) simulations was employed to investigate conformational dynamics and ligand binding within the M49 family (dipeptidyl peptidase III family). Six dipeptidyl peptidase III (DPP III) orthologues, human, yeast, three bacterial and one plant (moss) were studied. According to the results, all orthologues seem to be quite compact wherein DPP III from the thermophile *Caldithrix abyssi* seems to be the most compact. The protected regions are located within the two domains core and the overall flexibility profile consistent with semi-closed conformation as the dominant protein form in solution. Besides conservation of conformational dynamics within the M49 family, we also investigated the ligand, pentapeptide tynorphin, binding. By comparing HDX data obtained for unliganded protein with those obtained for its complex with tynorphin it was found that the ligand binding mode is conserved within the family. Tynorphin binds within inter-domain cleft, close to the lower domain β-core and induces its stabilization in all orthologues. Docking combined with MD simulations revealed details of the protein flexibility as well as of the enzyme–ligand interactions.

## Abstract 2

The hydrogen deuterium exchange (HDX) mass spectrometry combined with molecular dynamics (MD) simulations was employed to investigate conformational dynamics and ligand binding within the M49 family (dipeptidyl peptidase III family).

## Graphical abstract

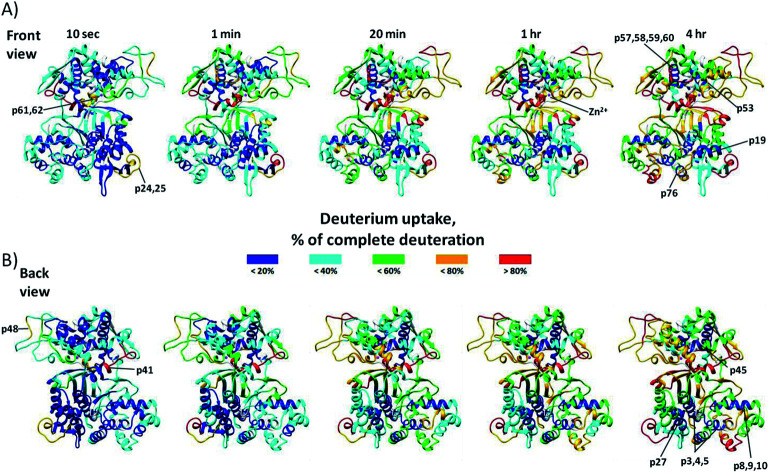

## Introduction

Dipeptidyl peptidase III (DPP III) enzymes belong to the M49 metallopeptidase family.^1^ To cleave dipeptides from the N-termini of various peptides consisting of three to ten amino acids, they need the zinc cation positioned within the active site.^2^ Human dipeptidyl peptidase III (hDPP III, UniProt entry: Q9NY33) can also be found labelled as enkephalinase B because it degrades neuropeptide enkephalin which takes part in pain regulation.^3,4^ Overexpression of hDPP III is observed in several pathophysiological conditions but its exact function is not entirely understood.^5–7^ Besides human DPP III, a number of the other M49 family orthologues, from eukaryotic (mammals,^1^ plants,^8^ insects,^9^ yeast^10^) and prokaryotic (bacteria^11,12^) organisms, have been identified. Their presence in different cells where they participate in the final steps of a protein catabolism indicates their importance for life.^13–17^ Until now several 3D structures of human DPP III (hDPP III) have been determined, both ligand free and in complexes with natural peptides (PDB_codes 3FVY and 5EGY and 3T6B, 3T6J, 5E33, 5E3A, 5E2Q, 5EHH and 5E3C, respectively).^18,19^ Additionally, the crystal structures for yeast and two bacterial orthologues (*Bacteroides thetaiotaomicron* and *Caldithrix abyssi*) were determined as well (PDB_codes: 3CSK, 5NA7 and 6EOM, respectively). Although the structures of all these DPP III orthologues have a characteristic M49 family fold organized in two domains, with an upper domain having a conserved motif of amino acids responsible for the zinc ion binding and catalytic function, separation, and orientation of domains significantly differ. An exception is a similarity between the unliganded yeast (yDPP III) and human DPP III (PDB_codes: 3CSK and 3FVY, respectively)^10,18^ structures with RMSD of 2.17 Å (calculated for 676 amino acids residues backbone) though their sequence identity is only about 35%. In these structures, domains are separated with a wide cleft so we call them open. In all crystal structures of hDPP III, except 3FVY, the domains are close to each other, and entrance to the active site is closed (PDB_codes: 5EGY, and 3T6B, 3T6J, 5E3A, 5E2Q, 5EHH and 5E3C).^19^ Computational studies have shown that the closed form is energetically more favorable for the catalytic reaction^20^ but it is too compact to allow the substrate molecule to enter the binding site. The molecular simulations showed that hDPP III protein in solution predominantly resides in semi-closed conformation.^21,22^ The crystal structures of the hDPP III in complexes with substrates and inhibitors revealed similar binding of peptides (substrates and inhibitors) with different amino acid sequences and length and suggested that the large domain motion induced by their binding is also conserved.^19^ This finding is in accord with a low specificity of DPP III enzymes towards the amino acid composition of peptide substrates.^11,23,24^ In the crystal structure of the hDPP III–tynorphin complex (PDB codes 3T6B and 3T6J), the ligand has an extended conformation and it forms several backbone hydrogen bonds with the five-stranded β-core of the lower domain. The N-terminus is also stabilized by the lower domain residues while the second peptide bond carbonyl is hydrogen bonded to His568 from the upper domain ensuring proper positioning of the cleaving peptide bond with respect to the catalytic zinc ion. However, differences in enzyme activity^25^ and inhibition^26,27^ observed for other DPP III orthologues indicate that the tynorphin binding mode, as well as overall enzyme flexibility, might be different in these complexes. In this study for the first time hydrogen deuterium exchange reaction (HDX) followed by mass spectrometry^28,29^ was applied to investigate the conformational dynamics conservation within the M49 (DPP III) enzyme family. HDX is effective method for localizing and determining protein stability and dynamics.^30^ Combined with molecular docking and dynamics simulations, it can be used for computer-aided drug design.^31^ Only a few peptidases have been studied using HDX approach. Most recently, dynamics and ligand-induced conformational changes in human prolyl oligopeptidase were analyzed by HDX.^32^ Our HDX experiments were performed for six orthologues: human (hDPP III), yeast (yDPP III), *Bacteroides thetaiotaomicron* (*Bt*DPP III), *Porphyromonas gingivalis* (*Pg*DPP III), *Physcomitrella patens* (*Pp*DPP III) and *Caldithrix abyssi* (*Ca*DPP III). The hexapeptide zinc binding motif (HEXXGH) was established as a “hallmark” of this family,^2^ required for the hydrolytic activity. However, in some new members of the M49 family, conserved hexapeptide motif is reduced to the pentapeptide HEXXH. Among the enzymes considered in this study, the DPP III orthologues from thermophile *C. abyssi* and moss *P. patens*, contain the pentapeptide motif. HDX flexibility map was obtained for each unliganded protein allowing localization of the conformational dynamics within the macromolecular sequence and the experimental value was mapped onto the protein 3D structure. Besides conformational dynamics of the ligand free peptidases, tynorphin binding into the interdomain cleft and its influence on the domain motion was investigated as well. By comparing the results of the HDX experiments with molecular modelling data we determined details of the enzyme–ligand interactions.

## Experimental methods

### Heterologous expression and purification

All DPPs III were expressed in *E. coli* as recombinant proteins with a C-terminal His_6_-tag and purified using affinity chromatography and size exclusion chromatography. Detailed expression protocols were described elsewhere.^8,12,25,26,33^

Protein purity was confirmed by SDS-PAGE according to Laemmli.^34^ Protein concentrations were determined by the Bradford method.^35^

### Determination of kinetic parameters

The *K*_m_ values for hydrolysis of fluorogenic substrate Arg_2_-2NA were determined at pH 7.4 (20 mM Tris–HCl buffer) and at 25 °C, from the initial reaction rates as previously described.^26^ Under the conditions used, the *K*_m_ values were 6.0 μM, 12 μM and 0.8 μM for human, yeast, and *B. thetaiotaomicron* DPP III, and 0.3 μM, 15.9 μM and 23.8 μM for *Porphyromonas gingivalis*, *Caldithrix abyssi* and *Physcomitrella patens* enzyme, respectively.

Enzyme inhibition studies with tynorphin were performed using the Arg_2_-2NA as a substrate, according to Baršun *et al.*^36^ The inhibition constant, *K*_i_, was calculated according to the equation:

1 *K*_i_ = *i*/(*v*_0_/*v*_i_ − 1)·*K*_m_/(*K*_m_ + *s*)

where *v*_0_ and *v*_i_ are the initial hydrolysis rates of Arg_2_-2NA (given in concentration *s*) catalyzed by DPP III in the absence and presence of inhibitor concentration (*i*), and *K*_m_ is the Michaelis constant.

### H/D exchange experiment

In the first step of the HDX analysis, optimization of digestion conditions and establishing a list of peptic peptides for all DPP III orthologues was carried out. With non-deuterated sample, a 5 μL aliquot of protein stock (between 35–50 μM in 20 mM Tris, pH 7.4) was diluted 10 times by adding 45 μL of 20 mM Tris, pH 7.4 (H_2_O reaction buffer). Next, the sample was acidified by mixing with 10 μL of 2 M glycine buffer, pH 2.5 (H_2_O stop buffer). The sample was subjected to online pepsin digestion using a 2.1 mm × 30 mm immobilized pepsin resin column (Porozyme, ABI, Foster City, CA) with 200 μL min^−1^ flow of 0.07% formic acid in water as a mobile phase. Digested peptides were passed directly to the 2.1 mm × 5 mm C18 trapping column (Acquity BEH C18 VanGuard precolumn, 1.7 μm resin, Waters, Milford, MA). Trapped peptides were eluted onto a reverse phase column (Acquity UPLC BEH C18 column, 1.0 × 100 mm, 1.7 μm resin, Waters, Milford, MA) using a 6% to 40% gradient of acetonitrile in 0.1% formic acid (flow 40 μL min^−1^) supplied by a nanoACQUITY Binary Solvent Manager. The total time of a single run was 13.5 minutes. All fluidics, valves, and columns were maintained at 0.5 °C using the HDX Manager (Waters, Milford, MA) except the pepsin digestion column, which was kept at 20 °C inside the temperature-controlled digestion column compartment of the HDX manager. The C18 column outlet was coupled directly to the ion source of a SYNAPT G2 HDMS mass spectrometer (Waters, Milford, MA) working in ion mobility mode. Lock mass was activated and carried out using leucine-enkephalin (Sigma). For protein identification, mass spectra were acquired in MSE mode over the *m*/*z* range of 50–2000. The spectrometer parameters were as follows: ESI positive mode, capillary voltage 3 kV, sampling cone voltage 40 V, extraction cone voltage 4 V, source temperature 80 °C, desolvation temperature 175 °C, and desolvation gas flow 600 L h^−1^. The spectrometer was calibrated on a weekly basis using standard calibration solutions. Peptides were identified using ProteinLynx Global Server software (Waters, Milford, MA). The list of identified peptides containing peptide *m*/*z*, charge, retention time, and ion mobility drift time was passed to the DynamX hydrogen deuterium data analysis program (Waters, Milford, MA).

Separate hydrogen deuterium exchange experiments were carried out for six unliganded DPP III enzymes, human V412I mutant, and their tynorphin complexes. The inhibition constant value (Table 1 ) was used to estimate proper tynorphin/enzyme ratio (*T*/*E*) present in stock solution in order to ensure that high percentage of the enzyme stays bound in complex under H/D exchange conditions. Those ratios were: 4.4 for hDPP III (99.76% bound); 3.3 for protein variant V412I hDPP III (99.91% bound); 10.7 for yDPP III (98.97% bound); 20 for *Bt*DPP III (98.98% bound); 11.1 for *Pg*DPP III (98.44% bound); 13.6 for *Pp*DPP III (98.60% bound) and 120 for *Ca*DPP III (97.26% bound). The reaction buffer was prepared using D_2_O (99.8% Armar Chemicals, Switzerland), in which the pH (uncorrected meter reading) was adjusted using DCl or NaOD (Sigma). After mixing 5 μL of protein stock with 45 μL of D_2_O exchange reaction was started at room temperature. Exchange reaction was started and followed for five time periods (10 s, 1 min, 20 min, 1 h and 4 h) each carried out in triplicate. The exchange reaction was quenched by reducing the pH to 2.5 by adding the reaction mixture to a plastic tube containing stop buffer (2 M glycine buffer, pH 2.5) cooled on ice. Immediately after quenching, the sample was manually injected into the nanoACQUITY (Waters, Milford, MA) UPLC system. Further pepsin digestion, LC, and MS analysis were carried out exactly as described for the non-deuterated sample. Relative deuterium uptake values are calculated without correction for back exchange. Peak deconvolution of the yeast DPPIII ^407^VRLKIGFKNVSLGNIL^422^ bimodal peak envelopes was carried out with MultiPeakFit package of the Igor Pro software (Igor Pro v 6.372, Multi-peak fit v2, Gauss fit, WaveMetrics, Inc.). Differential comparisons between HDX results for unliganded and tynorphin complex for each DPP III orthologue were carried out following the procedure described in Houde *et al.*^37^

**Table 1:** Inhibition of various DPP III orthologues by tynorphin

Enzyme	*K*_i_ tynorphin (μM)
hDPP III	0.03
yDPP III	0.47
*Bt*DPP III	0.98
*Pg*DPP III	0.72
*Pp*DPP III	0.66
*Ca*DPP III	16.75

**Table 2:** Deuterium uptake of the unliganded DPP III orthologues

	After 10 seconds	After 4 hours
	>30%	<20%
hDPP III	12%	18%
yDPP III	26%	15%
*Bt*DPP III	13%	22%
*Pg*DPP III	19%	25%
*Pp*DPP III	19%	25%
*Ca*DPP III	13%	16%

### Homology modelling

Since the 3D structures of the DPP III from *Porphyromonas gingivalis* (*Pg*DPP III) and from *Physcomitrella patens* (*Pp*DPP III) have not yet been determined experimentally, we resorted to comparative modelling. The sequences were retrieved from the UniProt database (http://www.uniprot.org, accession numbers: Q7MX92 and A9TLP4, respectively) and their structures were predicted using two approaches, the web server Phyre2 [http://www.sbg.bio.ic.ac.uk/∼phyre2]^38^ and the stand alone program Modeller9.^39,40^ In both cases models were built from two parts. The 3D structure of the DPP III domains were determined using the experimentally determined structures of *Bt*DPP III (PDB_code: 5NA7) and *Ca*DPP III (PDB_code: 6EOM), respectively. The sequence similarity between *Bt*DPP III and the DPP III domain of *Pg*DPP III is 51%, while between *Ca*DPP III and the DPP III domain of *Pp*DPP III it is 42%. In order to identify a suitable templates for the ARM and NUDIX domains a PSI-BLAST^41^ search was done. Following the multiple sequence alignment by Clustal Omega (https://www.ebi.ac.uk/Tools/msa/clustalo/) the 3D structures of *Pg*DPP III and *Pp*DPP III were determined using the program Modeller wherein for the ARM domain of *Pg*DPP III (660–886 aa residues) the 3D structure with PDB_code 3ZBO was used as a template, and for the NUDIX domain of *Pp*DPP III (aa 1-160 residues) the 3D structure with PDB_code 2FKB.

### Molecular dynamics (MD) simulations

MD simulations were accomplished for all ligand free DPP III orthologues as well as for their complexes with tynorphin, except for the *Pp*DPP III. In the case of human, yeast, *B. thetaiotaomicron* and *C. abyssi* DPP III, the crystallographically determined structures were used as initial structures while for the *P. gingivalis* and *P. patens* DPP III the initial structures were derived using homology modelling.^38^ The protein parametrisation was performed within the ff14SB^42^ force field using leap, a basic preparation program for Amber simulations available within the AMBER16 package (http://ambermd.org).^43,44^ For the zinc ion, Zn^2+^, parameters derived in previous work were used.^24^ All Arg and Lys residues in the structure were positively charged (+1e) while Glu and Asp residues were negatively charged (−1e), as expected at the physiological (experimental) conditions. The protonation of histidines was checked according to their ability to form hydrogen bonds with neighboring amino acid residues or to coordinate the metal ion.

The proteins and protein-substrate complexes were placed into a truncated octahedron box filled with TIP3P water molecules,^45^ and Na^+^ ions^46^ were added in order to neutralize the systems.

Before running productive molecular dynamics simulations, the protein geometry was optimized in three cycles (every 1500 steps) and the system was equilibrated. In the first cycle of optimization, water molecules were relaxed, while the rest of the system was harmonically restrained with a force constant of 32 kcal mol^−1^ Å^−1^. In the second and third cycle, the same force constant (32 kcal mol^−1^ Å^−1^) was applied to the zinc ion, while the protein backbone was restrained with force constants of 12 and 2 kcal mol^−1^ A^−1^, respectively. The energy minimization procedure, consisting of 470 steps of steepest descent followed by conjugate gradient optimization for the remaining steps, was the same in all cycles. During the first period of equilibration (200 ps of gentle heating from 0 to 300 K with a time step of 1 fs), the *NVT* ensemble was used, while all of the following simulations were performed at constant temperature and pressure (300 K and 1 atm, 2 fs time step, the *NpT* ensemble). During the equilibration, the zinc ion and/or its ligands were weakly restrained. The temperature was held constant using Langevin dynamics^47^ with a collision frequency of 1 ps^−1^. The pressure was regulated by a Berendsen barostat.^48^ Bonds involving hydrogen atoms were constrained using the SHAKE^49,50^ algorithm. The ligand free proteins for which the crystal structure was available were equilibrated for 1 ns and in the case of the initial structure derived by comparative modelling 50 ns of equilibration was performed. For each orthologue at least 150 ns of productive MD simulations was accomplished.

### Docking

In the case of hDPP III–tynorphin complex the crystal structure of the complex (PDB_code: 3T6B) was used as initial, while in the case of the other orthologues complexes were derived using the hDPP III–tynorphin complex as a template and the most appropriate orthologue structure generated during the simulations of the ligand free protein. As the most appropriate structure we assumed semi-closed enzyme form from the trajectory region with stable RMSD. Namely our earlier exhaustive investigations of the DPP III conformational search^21^ have indicated that the semi-closed enzyme are the most populated in water and therefore probably the most reactive state for substrate recognition. Thus obtained complexes were energy minimized and equilibrated using the same procedure as described above for the ligand free enzyme. The equilibrated complexes were simulated for at least 100 ns.

### Data analysis

The hydrogen bond analyses were performed with the CPPTRAJ module^51^ of the Amber16 program package.

## Results and discussion

### Conformational dynamics of the unliganded human DPP III

**Fig. 1:**
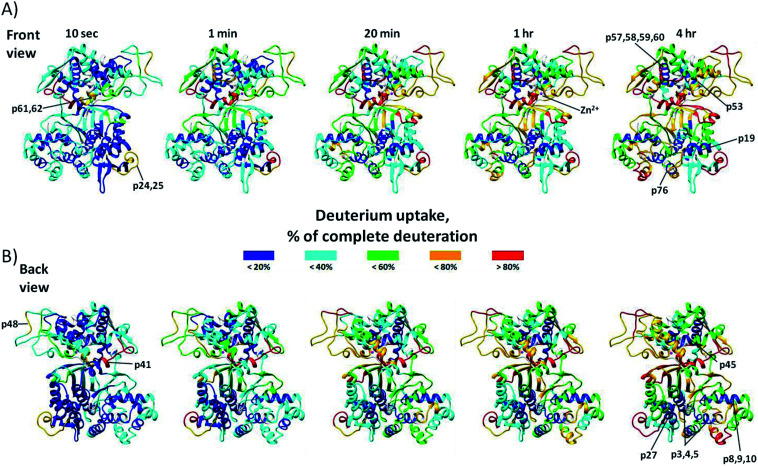
Conformational flexibility of unliganded hDPP III enzyme. Deuterium uptake values for the peptides are expressed as the percentage of the maximum incorporation measured in control experiment. Relative percentage values for each incubation time period are color-coded and mapped onto the crystal structure of the unliganded hDPP III enzyme (PDB_code 3FVY) and presented in front (A) and back view (B). Parts of the structure colored white are not covered with peptic peptides (detailed data about sequence coverage in Fig. S1A†). Protein structure regions discussed in the text are indicated. For the labeled peptides identification (sequences) see Table S1.†

Online pepsin digestion of the human DPP III protein showed excellent proteolytic efficiency due to the high enzyme/protein ratio and produced enough fragments to achieve 93.7% of the sequence coverage during 1.5 min digestion time (see Fig. S1A†). In human DPP III four highly flexible regions with fast H/D exchange were identified (Fig. 1): ^217^ASVLGSEPSLDSEVTSKLKS^236^ (p24 and p25), ^414^AVAYATQREKLTF^426^ (p41), ^557^YTPEAFNWRQAHM^569^ (p61 and p62), and ^470^NFDQE^474^ (p48). In these regions, located either in the loop of the hinge region or at the surface of upper and lower domain, more than 60% of amide hydrogens were exchanged within 10 seconds time period. After 4 hours long time period of H/D exchange several peptides exchanged less than 20% of their available amide hydrogens (Fig. 1). The protected regions are mostly located within α-helices of both enzyme domains: upper domain (^538^VIYVNWLNMVRAGLLALEF^556^ (p57, p58, p59 and p60), ^444^DVQVGL^449^ (p45), and ^510^RAESVGL^516^ (p53)) and lower domain (^33^YAYHLSRAAWYGGLAVLLQTSPEAPYIYAL^62^ (p3, p4 and p5), ^179^EDAKLAQD^186^ (p19), ^89^YQAFLVYAAGVYSNMGNY^106^ (p8, p9 and p10), ^256^QKVVEQL^262^ (p27), and ^714^ILTQL^718^ (p76)). The upper domain of the hDPP III appears to be more prone to deuterium incorporation than lower domain over 4 hours of the exchange period. Two catalytic motifs, ^450^HEXXGH^455^ and ^507^EECRAE^512^, important for the Zn^2+^ ion coordination are part of the upper domain and are located close or entirely in peptides ^450^HELLGHGSGKL^460^ (p46) and ^510^RAESVGL^516^ (p53) which are fragments of protected α-helices (see Fig. S1B†). Medium H/D exchange kinetics measured for peptides located on the surface of the binding cleft indicates that in solution hDPP III takes semi-open conformation as suggested by MD simulations.^22^ For all peptides, we measured binomial exchange profile (EX2 exchange regime) indicating that amide hydrogens visit exchange competent state many times before one hydrogen is replaced with deuterium.

In other words for all hDPP III peptides, local unfolding/folding is faster than it is their exchange reaction rate. Broadening of the peptide isotopic envelope due to deuterium incorporation follows a binomial distribution. Agreement between the measured deuterium uptake and the uptake determined from the amide hydrogen bond analysis during MD simulations (Fig. S2†) was obtained with a correlation coefficient of 0.766. Calculation of the theoretical values is carried out according to the similar procedure already published.^52^ Apart from the limitations of MD simulation, *i.e.* the conformations sampled during 150 ns present only a part of the protein conformational space, the correlation between the experimental and theoretical data depends on the quality of the HDX prediction model and approximations that are taken into account as well.^53–55^

### Structure flexibility conservation within DPP III family

Measurement of the localized hydrogen deuterium exchange data at different time intervals (from 10 seconds to 4 hours) allowed us to discern conformational dynamics at narrow regions of the enzyme structure and to identify the fast, medium and slow H/D exchanging peptides.

**Fig. 2:**
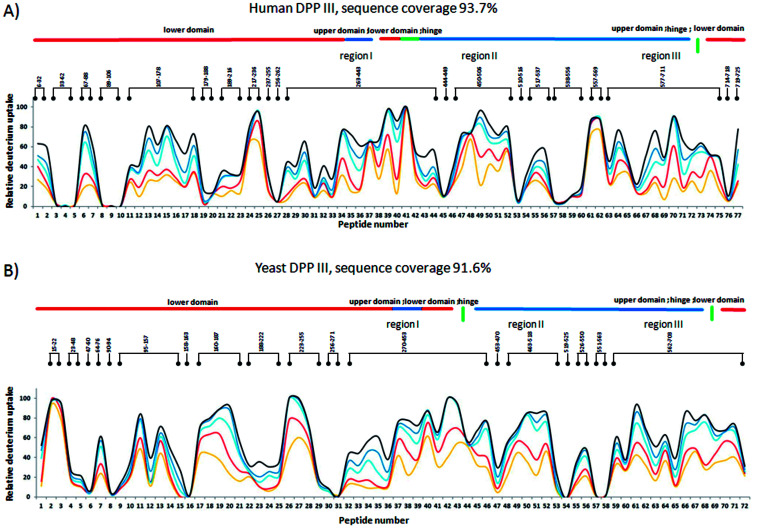
Fractional uptake of deuterium in peptides of (A) hDPP III, and (B) yDPP III, obtained by pepsin hydrolysis during five exposure periods: 10 s-yellow trace, 1 min-red trace, 20 min-light blue trace, 1 hour-dark blue trace and 4 hours-black trace. For the peptides identification (sequences), see Table S1.†

**Fig. 3:**
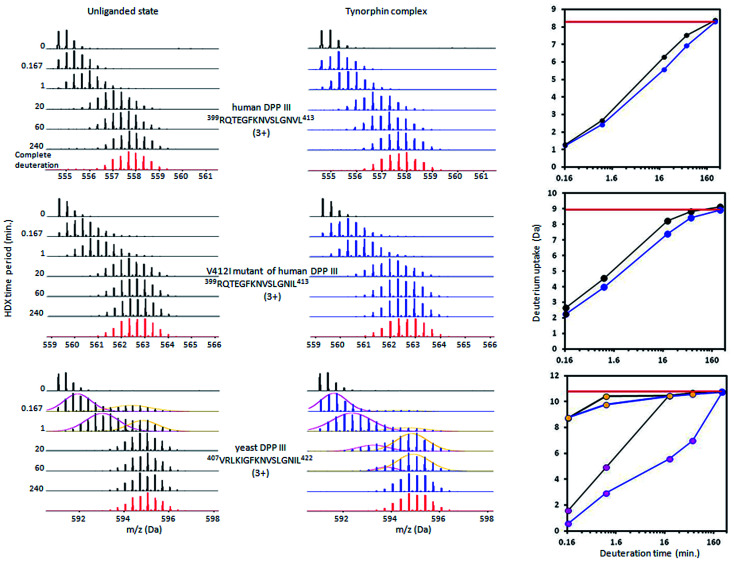
Deuterium uptake mass spectra for peptides covering hinge region within hDPP III, V412I hDPP III mutant and yDPP III. Mass spectra (left) are acquired at 10 s, 1 min, 20 min, 1 h and 4 h of incubation time for the unliganded and tynorphin complex state. Relative deuterium uptake plots (right) for the peptides kinetic curves in unliganded (black line) and in the tynorphin complex state (blue line). The complete deuteration is represented by red line. Isotopic envelope peaks for hDPP III ^399^RQTEGFKNVSLGNVL^413^ and for ^399^RQTEGFKNVSLGNIL^413^ from hDPP III V412I mutant show the binomial distribution pattern indicating the EX2 exchange regime, while the isotopic envelope peaks for ^407^VRLKIGFKNVSLGNIL^422^ from yeast DPP III shows complex profiles due to the mixed EX1/EX2 exchange regime. In the corresponding deuterium uptake plot (right) pink dots represent the slow exchanging conformer and yellow dots the fast exchanging conformer.

Peptides with deuterium uptake larger than 30% of their available amide hydrogens after the shortest (10 seconds) exchange time period indicate region permanently exposed to water or region that often becomes exposed to solvent under conformational changes of the protein. On the other hand, peptides having less than 20% of their available amide hydrogens exchanged with deuterium after the longest (4 hours) exchange time period correspond to the protected, hydrophobically shielded protein regions with amide hydrogens involved in the internal hydrogen bond network. The ratio of fast and slow exchanging peptides can be considered a measure of enzyme conformational plasticity. For human DPP III the fast exchanging peptides cover just 12% of the amino acid sequence. The comparable level of H/D exchange was determined for all unliganded DPP III orthologues wherein the largest share of the fast exchanging peptides was found in yeast DPP III (Table 2 ). More detailed insight into the local conformational behavior of the unliganded DPP III enzymes was provided by analyzing relative fractional uptake values for the peptic peptides. The values measured for human and yeast orthologues are shown in Fig. 2. It can be noticed that patterns of the peptides with high H/D exchange determined for these two enzymes are similar. The peptides with the highest exchange ratio are part of the protein regions that visit the exchange-competent state many times during the labelling periods. Region I includes the amino acid residues 263–443 and 270–453 in the hDPP III and yDPP III, respectively and comprises several secondary structure elements, equivalent in hDPP III and yDPP III wherein the largest (five strands) β-sheet defines the bottom of the active site cavity. This region also includes the hinge region and several helices. In hDPP III these are helices 17, 18 and 19 in the hairpin, and helices 14, 15, 16 in the lower domain. Region II covers the unstructured loop in the upper domain between amino acid residues His450 and Tyr506. Region III covers motifs located in the upper domain; the beta hairpin (E), helices 31, 32 and 33 and the hinge region between Arg669 and Val673; a second strand of the beta sheet (A), the beta hairpin (F) and helix 34 (see Fig. S1B†). Peptides with minimum exchange indicate the highly protected protein regions with the amide hydrogens involved in stable internal hydrogen bonds. Such regions are helices 2, 3, 6, 35 and partially helices 12 and 14 in the lower domain of hDPP III and helices 23, 25 and 27 comprising the upper domain core. The substantial diversity in the enzymes amino acid composition (Table S2 and Fig. S3†) gives an explanation for the variability of the HDX profiles determined for different members of the M49 enzyme family. As can be seen by comparing the flexibility profiles of different M49 orthologues (Fig. S4†) the highest similarity between the deuterium uptake determined in different orthologues is in the region denoted as Region I in hDPP III. This common HDX pattern is related to the DPP III inter-domain dynamics. By comparing the structure of the unliganded hDPP III with the hDPP III structure in its complex with tynorphin Bezerra *et al.*^18^ found that the long range conformational change of the protein structure could be described as domain rotation around the ‘peptide hinge’ ^409^LGNVLAVAYATQ^420^. For all DPP III orthologues the HDX study revealed EX2 exchange regime except for the yeast DPP III peptide ^407^VRLKIGFKNVSLGNIL^422^ which exhibited mixed EX1/EX2 kinetics (Fig. 3). This peptide comprises the hinge residues ^418^LGNIL^422^ corresponding to residues ^409^LGNVL^413^ of hDPP III. Bimodal broadening of the isotope envelope (Fig. 3) observed in this case indicates that inter-domain motion which exposes this peptide to interact with deuterated solvent is slower than chemical exchange rate of the exposed amide hydrogen's so that several of them exchange at once. Such behavior is opposite to that represented with binomial broadening (Fig. 3) were inter-domain motion is fast enough so that hDPP III peptide ^399^RQTEGFKNVSLGNVL^413^ visits exchange competent conformation many times before one amide hydrogen is exchanged. Replacement of valine at position 412 with more bulky isoleucine has not resulted with the EX1/EX2 HDX kinetics in the human V412I mutant. Namely, the peptide ^399^RQTEGFKNVSLGNIL^413^ bearing isoleucine instead of valine still exhibited binomial exchange profile (EX2 exchange regime, Fig. 3) although overall exchange was higher than it was for wild type hDPP III (Fig. 3). Conformational space sampled during MD simulation is close to fully folded protein form^56^ covering fast local structural fluctuations which correspond to peptides that follow EX2 HDX kinetics and have binomial isotopic envelope broadening. Yeast DPPIII ^407^VRLKIGFKNVSLGNIL^422^ is the only peptide in this study that was exhibiting mixed EX1/EX2 exchange kinetics indicating existence of population with different conformational states where one of them is populated as a result of larger inter-domain motion occurring on relatively slower timescale (EX1) which was not identified in MD simulation. The conformation of the peptide (VRLKIGFKNVSLGNIL) itself has not changed significantly during the simulations, but its accessibility to solvent did. Namely, we traced two different forms of its surrounding; one in which the peptide's backbone is mostly solvated with SASA of the peptide nitrogen atoms (Val407N–Leu422N) being 6.1 Å^2^ and the other in which it is more protected with SASA of N407–N422 of 1.6 Å^2^ (Fig. S5†). According to this result, we could assume that these two forms of yDPPP III are (more or less) equally populated in the solution during the HDX experiment.

### Tynorphin inhibition study, impact on the DPP III activity

Opioid pentapeptide tynorphin (VVYPW) has been shown to be a potent inhibitor of DPP III hydrolytic activity.^57^ Earlier investigations suggested that the ligand binding into the inter-domain cleft influences the large domain motion and induces striking compression of human and bacterial DPP III orthologues.^18,19,21,58^ We assumed that comparative (differential) H/D exchange study on ligand free enzymes and their complexes with tynorphin would enable us to reveal the degree of conservation of the ligand binding site and influence of the ligand, tynorphin, binding on the enzyme flexibility.

The kinetic measurements of the enzyme inactivation by tynorphin revealed the different degree of inactivation in studied orthologues. As shown in Table 2, all six studied DPP III enzymes were inhibited by tynorphin. However, inhibitory potency differed significantly. Human DPP III was the most inhibited while four enzymes yDPP III, *Bt*DPP III, *Pg*DPP III and *Pp*DPP III have had inhibition constant values 10–30 times higher, and the *K*_i_ value for *Ca*DPP III was 500-fold increased, compared to hDPP III.

### Influence of the tynorphin binding to DPP III flexibility

**Fig. 4:**
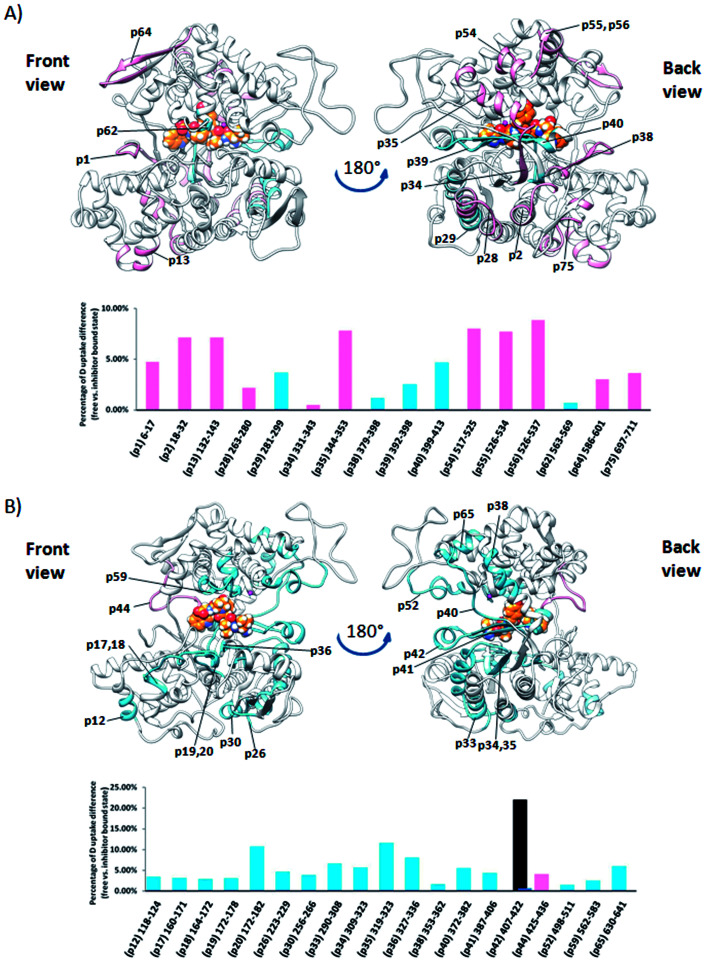
Graphical representation of the deuterium uptake differences found for peptic peptides of human and yeast DPP III by comparing two states, unliganded enzyme and its tynorphin complex. Presented are statistically significant values calculated as the difference in the area under deuterium uptake curves of two protein states (unliganded enzyme and its tynorphin complex) which is normalized to a maximal possible deuterium uptake curve area for that specific peptide. All differences are shown in Fig. S6.† Light blue bars denote positive differences, meaning that area under uptake curve was larger for unliganded enzyme than for its complex with tynorphin. Positive difference colored black is for slow exchanging conformer and dark blue for fast exchanging conformer. Pink bars denote negative differences indicating that deuterium uptake in the region of protein structure covered by the corresponding peptide was higher upon tynorphin binding. Such color-coded values for the labelled peptides are mapped onto a corresponding enzyme–tynorphin structure. (A) Ribbon representation of the hDPP III structure bound to the tynorphin inhibitor from a front (left) and back (right) view of the active site cleft. (B) Ribbon representation of the yDPP III structure bound to the tynorphin inhibitor from a front (left) and back (right) view of the active site cleft. In both structures, tynorphin was presented by colored sphere atoms (orange-C, white-H, red-O and blue-N). For the peptides' identification (sequences), see Table S1.†

**Fig. 5:**
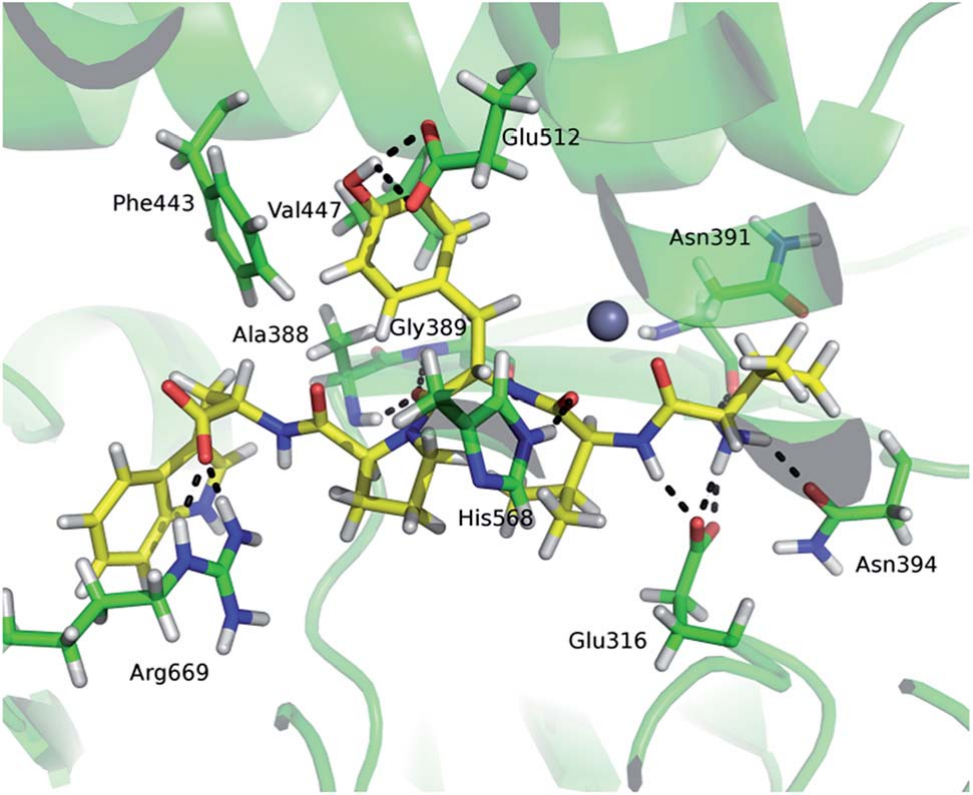
The tynorphin (carbon atoms are shown in yellow) binding in hDPP III obtained by MD simulations. Selected amino acid residues interacting with the substrate are shown (carbon atoms are shown in green). The intermolecular hydrogen bonds are represented by black dashed lines.

Comparison of the deuterium uptake by the matching peptides in unliganded hDPP III and its complex with tynorphin enabled us to localize changes in HDX rate upon tynorphin binding and to investigate the influence of ligand binding on the protein conformation and dynamics (Fig. 4A). In the crystal structure of the hDPP III complex tynorphin is bound in the form of a β-strand to the five-stranded β-core of the lower domain in an antiparallel fashion.^18^ In agreement with such a binding mode the four peptides of the hDPP III lower domain (^281^YIESFTQGSIEAHKRGSRF^299^ (p29), ^379^LTFAGSGIPAGINIPNYDDL^398^ (p38), ^392^IPNYDDL^398^ (p39), and ^399^RQTEGFKNVSLGNVL^413^ (p40)) as well as the peptide ^563^NWRQAHM^569^ (p62) from the upper domain, all in close contact with the bound tynorphin, underwent a decrease in deuterium uptake. Apparently, the ligand hinders the access of deuterated water to their amide hydrogens. Furthermore, an increase of the deuterium uptake upon ligand binding was measured at several regions distant from the active site (Fig. 4A) indicating conformational changes in these regions upon ligand binding. These regions are covered with the following peptides: ^18^DCREAFRLLSPTERL^32^ (p2); ^132^AAQQHPEEVRGL^143^ (p13), ^331^FVAVVNKAMSAKF^343^ (p34) and ^526^EIFGFEGADAED^537^ (p55) (Fig. 4A). These results are in agreement with the rearrangement of the protein intermolecular hydrogen bonds in these regions and their increased solvent accessibility noticed during the inter-domain conformation changes induced by ligand binding (Table S3†). During MD simulations of the human DPP III–tynorphin complex the ligand remained bound in an antiparallel fashion to a five-stranded β-core from the lower protein domain. The representative orientation of the ligand is shown in Fig. 5. Plots of the intermolecular hydrogen bonds lengths changes during MD simulations, as well as overlay of the starting and final conformation are given in Fig. S7.†

It is interesting to notice that most of the peptides showing an increase of the deuterium uptake upon ligand binding, like p2, p34 and p55/p56, are situated on the “back”, convex, side of the protein for which MD simulations had already reported^21^ the increase in residue based solvent accessible surface area during protein closure. Agreement with experiment was also obtained for the peptides p13, p34 and p55/p56 for which we have determined smaller mean number of hydrogen bonds per residue in the liganded enzyme structure than in the unliganded one (Table S3†). Consequently, the same four peptides from the lower domain (p29, p38, p39 and p40) and p62 peptide from the upper hDPP III domain interact with tynorphin and establish larger mean number of hydrogen bonds per residue in the complex structure than in the ligand-free structure obtained by the MD simulations (Table S3†).

**Fig. 6:**
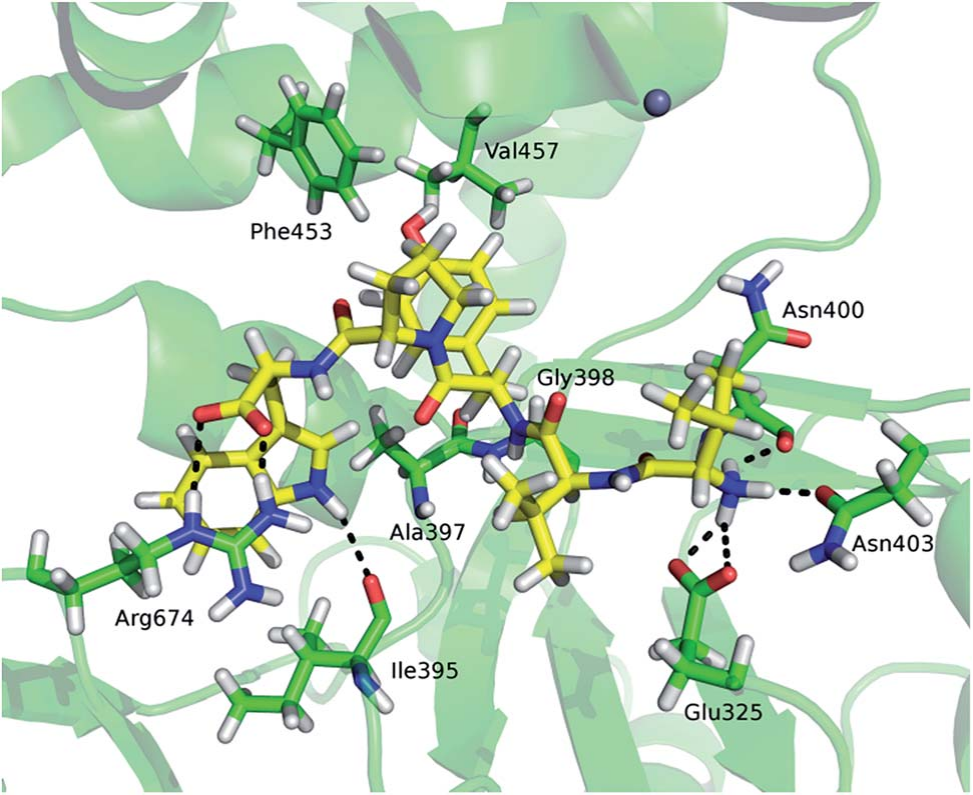
The tynorphin (carbon atoms are shown in yellow) binding in the yDPP III obtained by MD simulations. Selected amino acid residues interacting with substrate are shown (carbon atoms are shown in green). The intermolecular hydrogen bonds are represented by black dashed lines.

Although tynorphin binding did not induce the deuterium uptake increase in any region of yDPP III, its binding induced the deuterium uptake decrease in the upper part of the lower domain five-stranded β-core (Fig. 4B) as it was determined in the case of hDPP III. We assumed that the tynorphin binding modes in human and yeast DPP III are similar and built the yDPP III–tynorphin complex accordingly. During 100 ns of MD simulations the tynorphin remained bound in the form of a β-strand into the enzyme active site, close to the lower domain β-core. The representative orientation of the ligand is shown in Fig. 6, and the RMSD plot of the tynorphin backbone, as well as the overlay of three conformations generated during MD simulations are given in Fig. S8.† This is in agreement with the HDX results which revealed deuterium uptake decrease in the peptides from the predicted tynorphin binding site: ^290^YINHFVTGSSQAHKEAQKL^308^ (p33), ^372^YEKPIFNPPDF^382^ (p40), ^387^VLTFTGSGIPAGINIPNYDD^406^ (p41), ^407^VRLKIGFKNVSLGNIL^422^ (p42). Peptide p42 exhibits bimodal exchange kinetics and deuterium incorporation decrease in tynorphin complex. Decrease of less than 1% was measured for fast exchanging conformation indicating very weak protection from tynorphin. For slow exchanging conformation deuterium incorporation decrease is more than 22% indicating much stronger protection of the bound tynorphin (Fig. 3 and 4B).

Beside the ligand binding region, the reduced deuterium uptake was also observed in several other peptides from both lower domain: ^160^IGIYHVEEKAAL^171^ (p17), ^172^LGFPSQGYTSA^182^ (p20), ^223^QIWVASE^229^ (p26) and the upper domain: ^498^YKVGETWGSKFGQL^511^ (p52) and ^630^YLKHLHVYKCSG^641^ (p65). MD simulations revealed that ligand binding boosts the protein closure, *i.e.* the radius of gyration (Rgyr) of yDPP III decreases faster during MD simulation of the complex with tynorphin than of the ligand free protein (Fig. S8†), indicating that these peptides are either approaching the bound substrate (p52) or protein residues from the other domain (p17 and p20) in the yDPP III–tynorphin complex.

Differential HDX experiment with the human V412I mutant was carried out to check the importance of the amino acid composition within the hinge region for the conformation dynamics of the complex. By changing valine at position 412 in hDPP III to isoleucine the same amino acid composition in the hinge region of human (^403^GFKNVSLGNVL^413^) and yeast (^412^GFKNVSLGNIL^422^) orthologue was achieved. The differential HDX data analysis shows that tynorphin binding reduces deuterium uptake within the active site cavity in the regions of the V412I mutant which are getting in close contact with the inhibitor. Those are the parts of the β-core structure covered by peptides: ^285^FTQGSIEAHKRGSRF^299^ (p29), ^310^SYIGF^314^ (p31), ^312^IGFIES^317^ (p32), ^381^FAGSGIPAGINIPNYDDL^398^ (p38) and ^399^RQTEGFKNVSLGNIL^413^ (p40). Similarly to differential HDX experiment for yDPP III protein, the reduced deuterium uptake was observed for the upper domain peptide ^489^YRSGETWDSKF^499^ (p51) and for the lower domain peptide ^152^FSLEPRLRHLGLGKEGITT^170^ (p16) which are brought closer to each other by conformational change during the transition from open into the closed conformation. The position of these two peptides suggests that in the closed conformation the two domains are placed relative to each other like it was observed for yDPP III complex indicating a significant impact of the amino acid composition of the hinge region to the mode of closure and the inter-domain position. The H/D exchange curve observed for the mutant p40 peptide (Fig. 3) indicates faster deuterium exchange in the V412I mutant than in the wild-type hDPP III. This finding together with the absence of deuterium uptake increase in mutated enzyme suggests that the protein closure upon ligand binding is less pronounced in the V412I mutant than in the wild-type hDPP III.

**Fig. 7:**
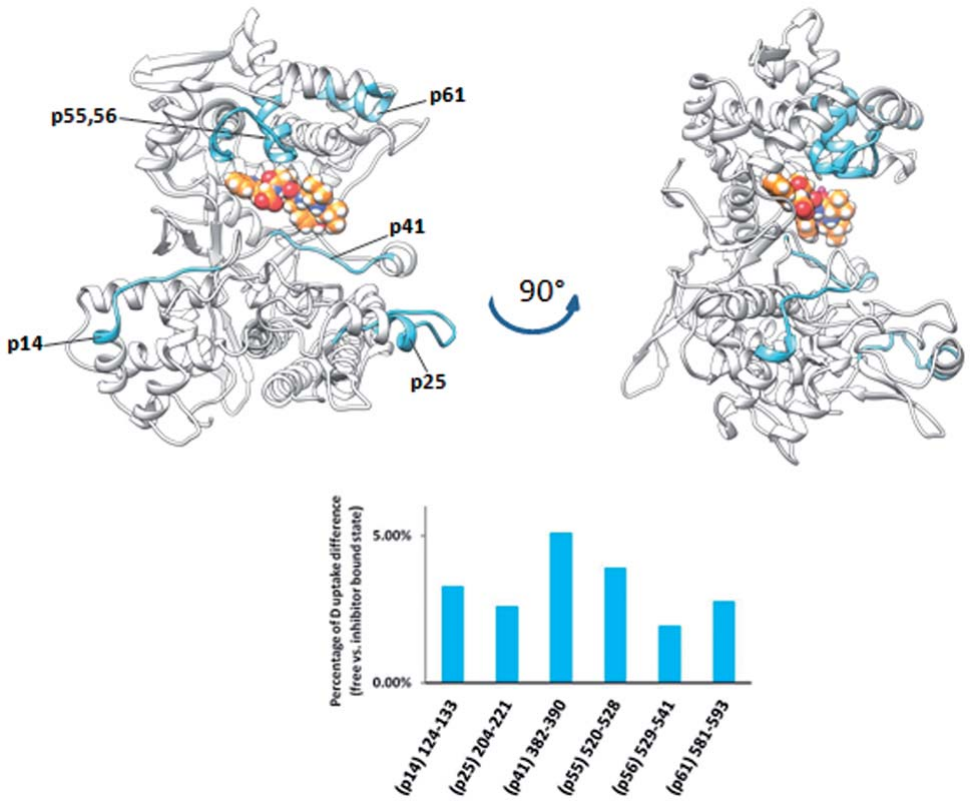
Graphical representation of the deuterium uptake differences found by comparing two states of the *Bt*DPP III (unliganded enzyme and its complex with tynorphin). Ribbon representation of the *Bt*DPP III structure (PDB accession number 5NA7) bound to the tynorphin inhibitor from a front (left) and side (right) view of the active site cleft is shown. For the peptides identification (sequences), see Table S1.†

**Fig. 8:**
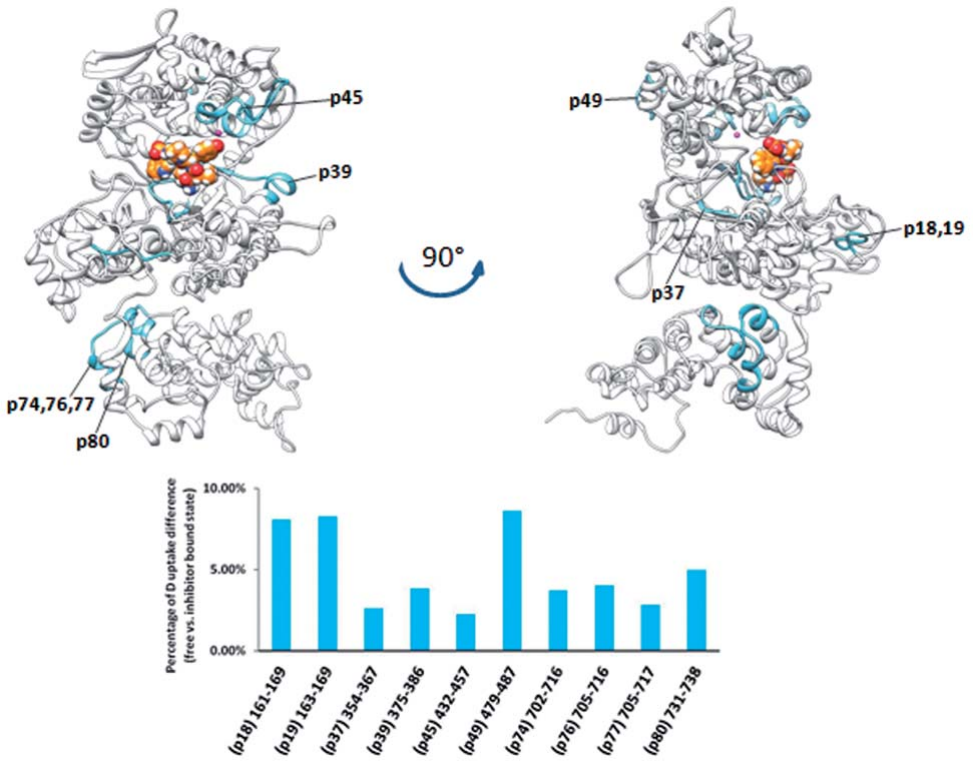
Graphical representation of the deuterium uptake differences found by comparing two states of the *Pg*DPP III (unliganded enzyme and its complex with tynorphin). Ribbon representation of the modelled *Pg*DPP III structure bound to the tynorphin inhibitor from a front (left) and side (right) view of the active site cleft is shown. For the peptides identification (sequences), see Table S1.†

Differential analysis of the HDX data obtained for two bacterial DPP III homologs, from *Bacteroides thetaiotaomicron* and *Porphyromonas gingivalis*, revealed only a weak influence of the tynorphin binding on the HDX kinetics (Fig. 7 and 8). Such findings are in agreement with the recently published crystal structure of the ligand free *Bt*DPP III, which is significantly more compact than human DPP III,^59^ and MD simulations results of the *Bt*DPP III which showed that the amplitude of the interdomain separation in hDPP III is significantly larger than in *Bt*DPP III.^58^ In the case of *Bt*DPP III the slight decrease in the deuterium uptake was observed for three peptides, ^382^IGINLPNAN^390^ (p42), ^520^LVRIEPGNN^528^ (p56) and ^204^YGAMKDPKDETPVSY^218^ (p26). The first is part of the lower domain beta strand and a loop within the active site cavity while the other two are located in the upper and lower domain, respectively. In accord with the observed decrease in deuterium uptake, molecular dynamics simulations of the *Bt*DPP III–tynorphin complex showed the substrate interactions with the amino acids from peptides p42 and p56 (Fig. S9 and Table S4†). The peptide p26 consists mostly of a single loop, which makes it susceptible to large conformational motions. During the *Bt*DPP III–tynorphin complex closure it translocated and established hydrogen bonds with the upper domain amino acids. Similar behavior has been previously reported for the *Bt*DPP III–Arg_2_-2NA complex.^58^ However, this conformational transition has not been traced during MD simulations of the ligand-free enzyme (Fig. S10†).

*Pg*DPP III is significantly larger (886 amino acid residues) than human, yeast, and *B. thetaiotaomicron* DPP III. It has all the evolutionarily conserved regions of the DPP III family but, differently from the other characterized DPP III orthologues, it possesses a C-terminal extension containing an armadillo (ARM) type of fold similar to that of the AlkD family of bacterial DNA glycosylases.

However, complementation assays in a DNA-repair-deficient *Escherichia coli* strain indicated the absence of alkylation repair function of this enzyme. On the other hand, its peptidase activity is comparable to that of *Bt*DPP III.^33^ Weak changes of the HDX profiles upon the enzyme complexation with tynorphin were detected in the DPP III part of the protein region represented by peptides p18, p37, p39, and p45, (^161^IIKASSVNF^169^, ^355^LTIAGDSYPATPIG^365^, ^375^WIRAEHGSKSVT^386^, and ^432^HECLGHGSGQLLPGVPGDALGEHAST^457^, respectively) and in the peptide p74 from the ARM fragment (Fig. 8). According to the computational results, the peptides p37 and p45 interact directly with peptide ligand during MD simulations of the *Pg*DPP III–tynorphin complex (Fig. S11 and Table S4†). Like in the case of human and yeast orthologue, ligand binding into the enzyme active site boosts the protein closure.^12^ In the case of *Pg*DPP III this resulted in concurrent movements of both the lower DPP III domain and ARM fragment in the direction of the upper DPP III domain as it was discussed in our recent publication.^33^ This could serve as an explanation for the measured HDX decrease in the regions distant from the tynorphin binding site.

**Fig. 9:**
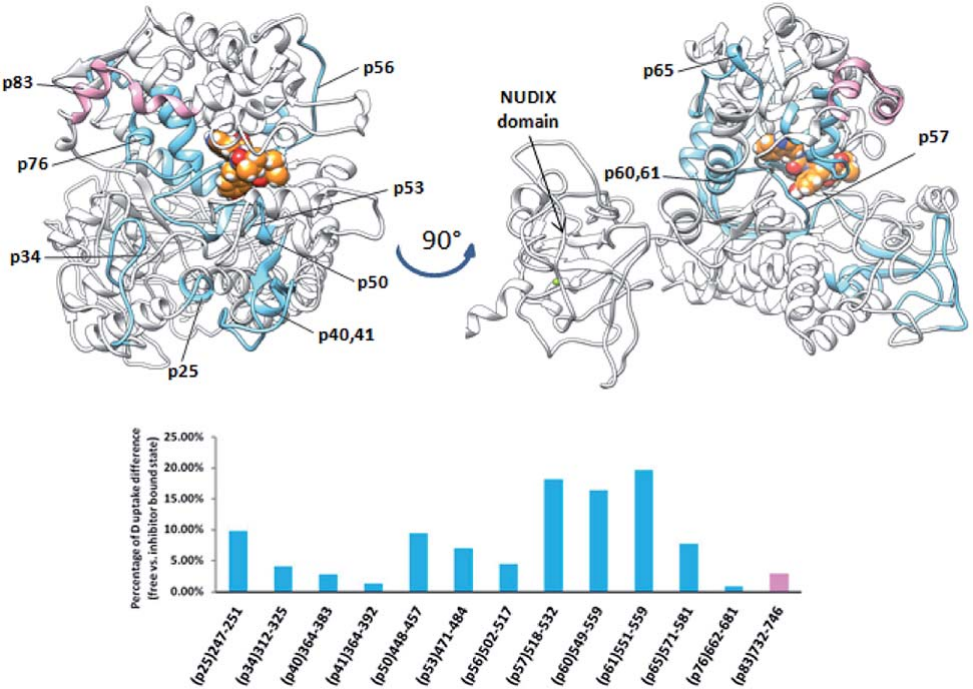
Graphical representation of the deuterium uptake differences found by comparing two states of the *Pp*DPP III (unliganded enzyme and its complex with tynorphin). Ribbon representation of the modelled *Pp*DPP III structure bound to the tynorphin inhibitor from a front (left) and side (right) view of the active site cleft is shown. For the peptides identification (sequences), see Table S1.†

The plant DPP III from moss *Physcomitrella patens* (*Pp*DPP III), differently from the other DPP III orthologues, contains the so-called NUDIX motif on the N-terminal part of the sequence, a characteristic of Nudix hydrolases (Fig. 9).^8^ Changes in HDX kinetics were detected only in part of the sequence covering DPP III domains, but not in the NUDIX domain (Fig. 9 and S6E†). All peptides with the reduced deuterium uptake upon complexation are located within the active site cleft indicating that tynorphin interacts with larger part of the beta core from the lower domain and so significantly reduces conformational dynamics of the active site cleft region.

Peptides with reduced deuterium uptake, p50, p53, p57, p60 and p65 (^448^VTIGPYETYE^457^, ^471^IGIRDDEATQRLKL^484^, ^518^LYNSGDVKGPQTVAF^532^, ^549^VMLKNISQAKF^559^ and ^571^VEASQRGAVDF^581^, respectively) contain amino acid residues from the ligand binding subsites and according to the molecular modelling results they take part in ligand stabilization (Fig. S12†).

**Fig. 10:**
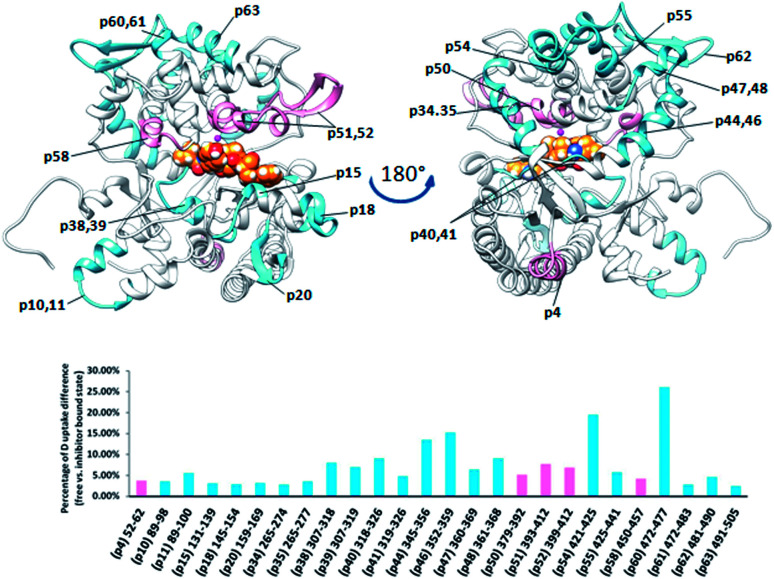
Graphical representation of the deuterium uptake differences found by comparing two states of the *Ca*DPP III (unliganded enzyme and its complex with tynorphin). Ribbon representation of the *Ca*DPP III structure bound to the tynorphin inhibitor from a front (left) and back (right) view of the active site cleft is shown. For the peptides identification (sequences), see Table S1.†

Comparing the HDX data obtained for unliganded DPP III from *Caldithrix abyssi* (*Ca*DPP III) and its complex with tynorphin revealed significant changes of the HDX kinetics in the upper domain induced by tynorphin binding. Even though the β-sheet in the lower domain was again identified as a potential substrate binding place (moderately small decrease of the deuterium uptake was observed in one peptide covering part of the beta strand ^307^SAGDTKAGVQTLA^319^ (p39)) the significant decrease in the deuterium uptake was found in distant peptides of the upper domain: ^345^AKFDKLLKPIAE^356^ (p44), ^351^LKPIAE^356^ (p45), ^352^KPIAEKVL^359^ (p46), ^361^AEQLPLVT^368^ (p48), ^421^YNNLF^425^ (p54), ^425^FMIEKGVYPPEFEKQIY^441^ (p55) and ^472^LEKGAY^477^ (p60) (Fig. 10).

**Fig. 11:**
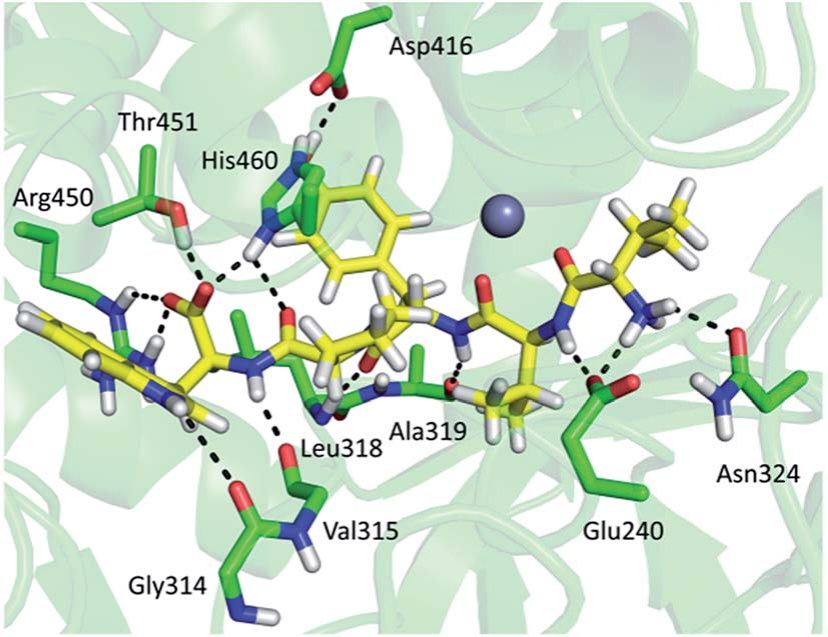
Binding of the tynorphin (carbon atoms are shown in yellow) in the *Ca*DPP III obtained by MD simulations. Selected amino acid residues interacting with substrate are shown (carbon atoms are shown in green).

The other three peptides ^379^HEISHGLGPGKIVL^392^ (p50), ^393^NGRQTEVKKELKETYSSIEE^412^ (p51) and ^450^RTIRFGIN^457^ (p58) in the *Ca*DPP III upper domain show an increase in deuterium uptake while the peptide ^89^RASSDPLDQLRL^100^ (p11), covering a small region in lower domain, experienced a decrease in deuterium uptake upon complexation. In summary, the binding of tynorphin significantly changes flexibility of the upper domain of *Caldithrix abyssi* DPP III. It should be noted that peptides p50 and p51 comprise the amino acids from the conserved regions, pentapeptides HEISH and EECK(R)A. MD simulations revealed a decrease of the H-bond population upon the substrate binding in the regions comprising peptides p50, p51 and p58, clearly showing that the tynorphin binding destabilizes the catalytically relevant amino acids (see Table S5† for the hydrogen bond population in the relevant peptides). However, like in the mesophylic orthologues, tynorphin binds in the form of a β-strand into the *Ca*DPP III active site, close to the lower domain β-core (Fig. 11).

Both X-ray diffraction and MD simulations showed that the structure of the ligand free *Ca*DPP III is much more compact than the ligand free structures of the other DPP III orthologues, which might be the reason for the significantly higher *K*_i_ value determined for tynorphin.^12,18,19,59^

## Conclusions

In this work, we investigated conservation of the conformational flexibility within the DPP III enzyme family using HDX-MS experiments combined with MD simulations. Altogether, 6 DPP III orthologues have been studied: human, yeast, three bacterial and moss.

Inspection of the H/D exchange in conserved protein regions revealed that the region containing the hexapeptide (or pentapeptide in the case of *Ca*DPP III and *Pp*DPP III) signature motif is, in general, among the most protected, *i.e.* the most rigid, regions within the considered orthologues, with exception of *Ca*DPP III. The relative protection is highest in the human orthologue, in agreement with its high activity. In addition, results of H/D exchange experiment agree with the results of MD simulations which showed that in solution the semi-closed human DPP III form is the most populated one.

The similarities in the human and yeast DPP III flexibility profiles are closely correlated with the similarity of their 3D structures (RMSD of about 2 Å).

Besides the flexibility conservation, we also studied the possible differences in the ligand, pentapeptide tynorphin, accommodation into the enzyme and its influence on the protein conformation and local flexibility.

Despite differences in orthologues' structure and flexibility, we found that in all cases tynorphin binds to the upper part of the lower domain β-sheet.

It seems that the more structured enzymes (*i.e.* enzymes with small share portion of unstructured regions like loops), such as human and *C. abyssi* DPP III, are more sensitive to ligand binding than those whose structure is more disordered, *e.g. Bt*DPP III and *Pg*DPP III. The tynorphin binding mode previously determined in human DPP III by X-ray diffraction is, according to the differential H/D exchange study and MD simulations, preserved in complexes with different DPP III orthologues.

## Conflicts of interest

There are no conflicts of interest to declare.

## Supplementary Material

RA-008-C7RA13059G-s001
