# Supplementary material for: Conservation of the conformational dynamics and ligand binding within M49 enzyme family
Source: RSC Adv. 2018 Apr 10;8(24):13310–22. doi: 10.1039/c7ra13059g (PMC9079729; doi:10.1039/c7ra13059g)
Supplement: RA-008-C7RA13059G-s001 [file RA-008-C7RA13059G-s001.pdf]

Electronic Supplementary Material (ESI) for RSC Advances.
This journal is © The Royal Society of Chemistry 2018

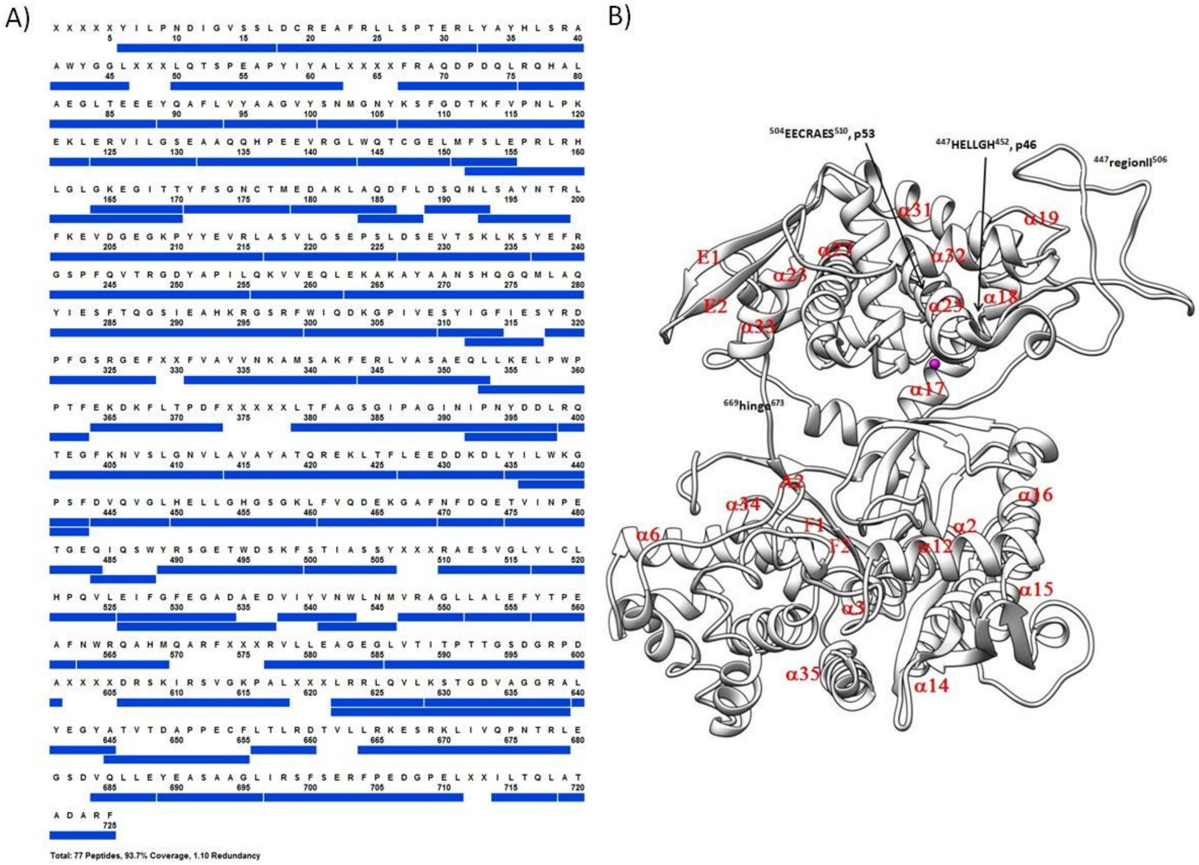

Figure S1. A) Human DPP III enzyme sequence coverage map covering 93.7% of the entire sequence with 77 peptic peptides. B) Elements of secondary structure for which hydrogen deuterium kinetics is discussed are shown mapped onto 3D structure of the unliganded human DPP III enzyme (PDB accession number 3FVY).

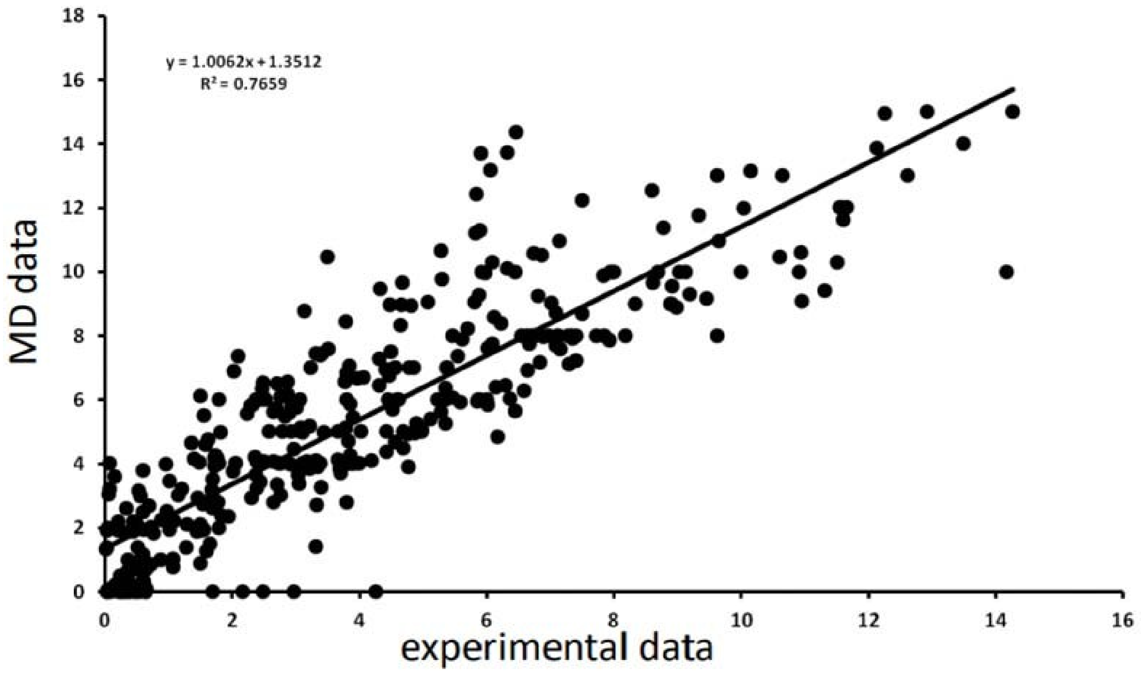

Figure S2. Deuterium uptake correlation for all unliganded hDPP III peptides at all time periods between experimental values and values estimated by MD based H-bond statistics as described in Park et al. (I.-H. Park, J. D. Venable, C. Steckler, S. E. Cellitti, S. A. Lesley, G. Spraggon and A. Brock, *Journal of Chemical Information and Modeling*, 2015, **55**, 1914-1925.)

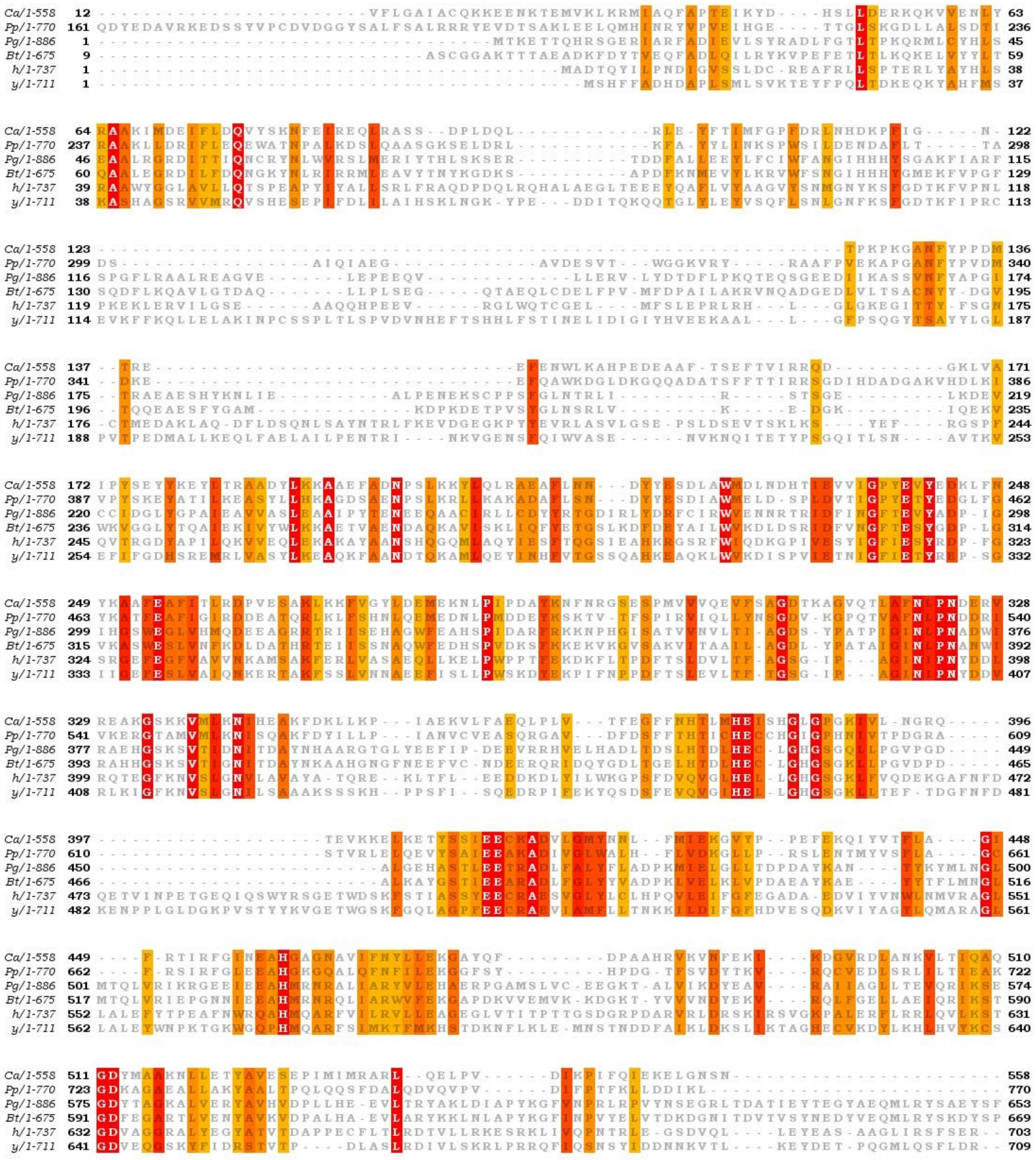

Figure S3. Multiple alignment of DPP III orthologs' sequences. Shown are sequences of *Caldithrix abyssi* (UniProt KB: H1XW48), *Physcomitrella patens* (UniProt KB: A9TLP4), *Porphyromonas gingivalis* (UniProt KB: Q7MX92), *Bacteroides thetaiotaomicron* (UniProt KB: Q8A6N1), *Homo sapiens* (UniProt KB: Q9NY33) and *Saccahromyces cerevisiae* (UniProt KB: Q08225).

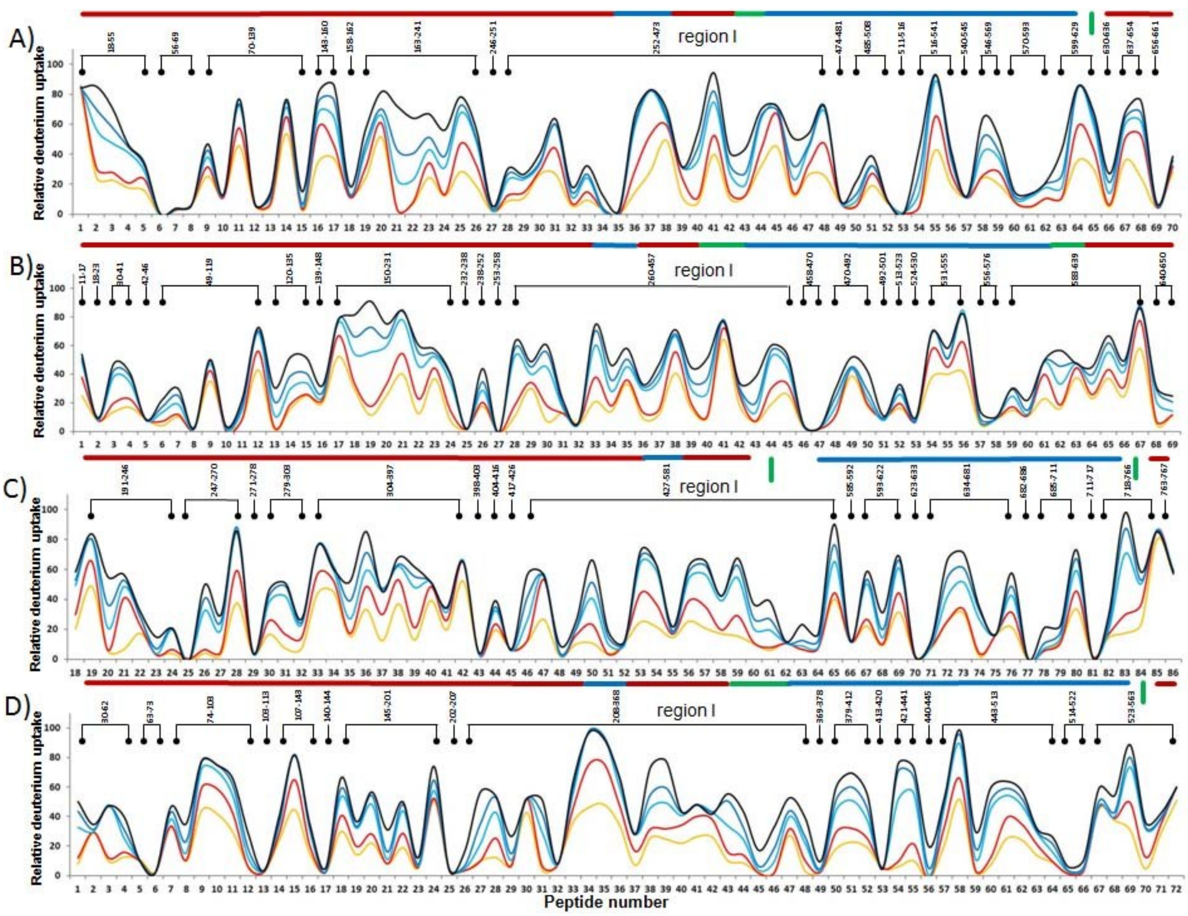

Figure S4. Fractional uptake of deuterium in peptides of A) *Bt*DPP III, B) *Pg*DPP III, C) *Pp*DPP III and D) *Ca*DPP III, obtained by pepsin hydrolysis during five exposure periods: 10s-yellow trace, 1 min-red trace, 20 min-light blue trace, 1 hour-dark blue trace and 4 hours-black trace. Values are not corrected for back exchange. For the peptides identification (sequences), see Table S1

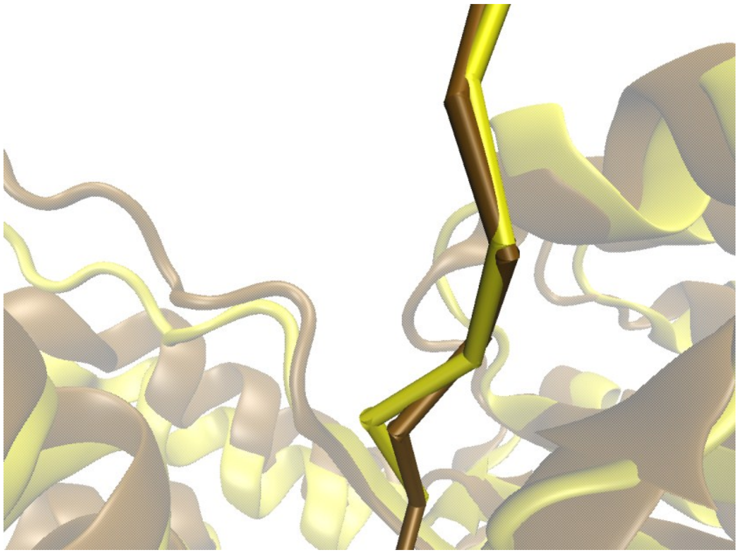

Figure S5 Overlay (superposition) of two yDPP III conformers in which peptide VRLKIGFKNVSLGNIL, for which binomial behaviour was found, is less (ochre) and more (yellow) solvent exposed

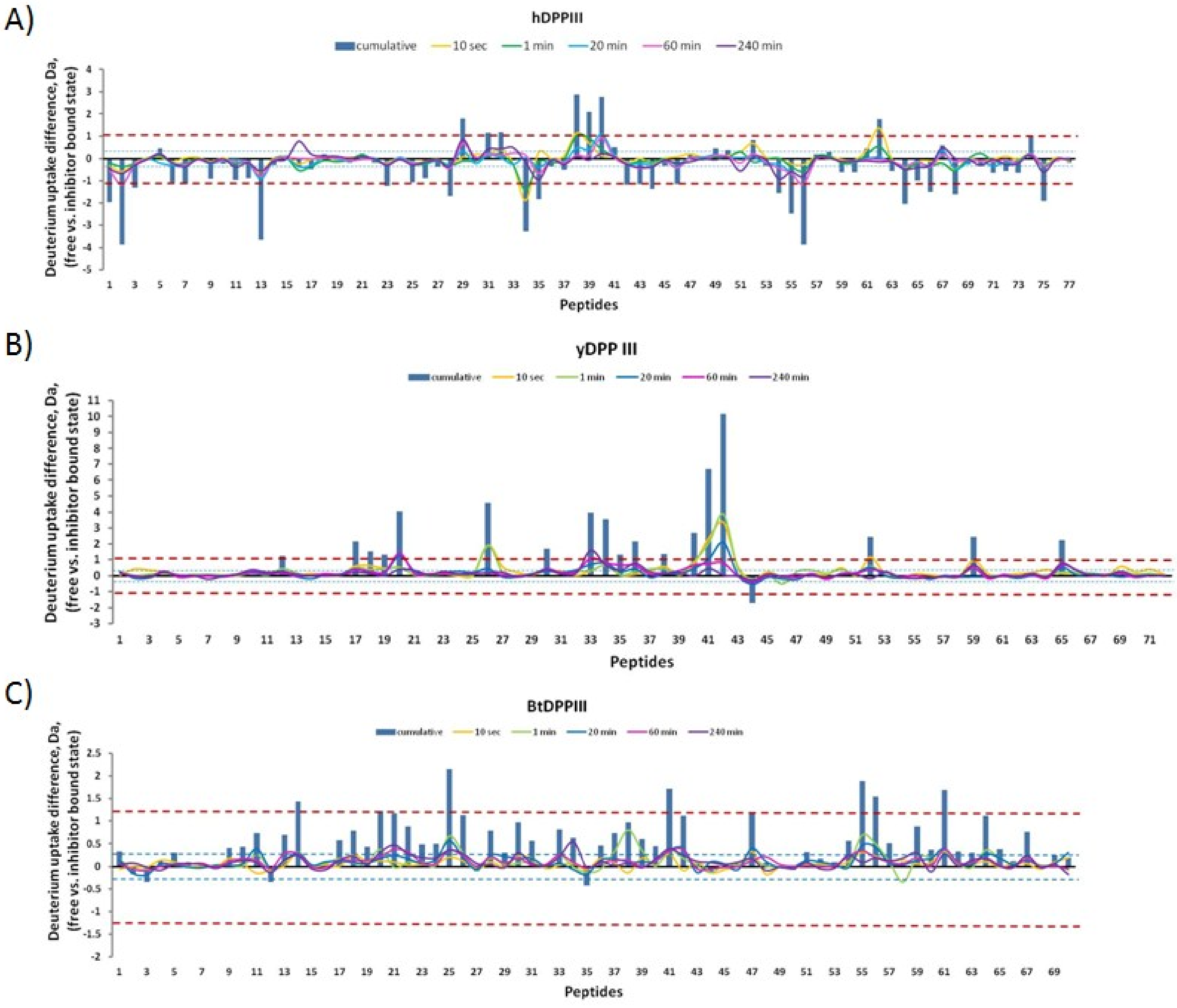
A)
hDPPIII
cumulative
10 sec
1 min
20 min
60 min
240 min
Deuterium uptake difference, Da, (free vs. inhibitor bound state)
Peptides
B)
yDPP III
cumulative
10 sec
1 min
20 min
60 min
240 min
Deuterium uptake difference, Da, (free vs. inhibitor bound state)
Peptides
C)
BtDPPIII
cumulative
10 sec
1 min
20 min
60 min
240 min
Deuterium uptake difference, Da, (free vs. inhibitor bound state)
Peptides

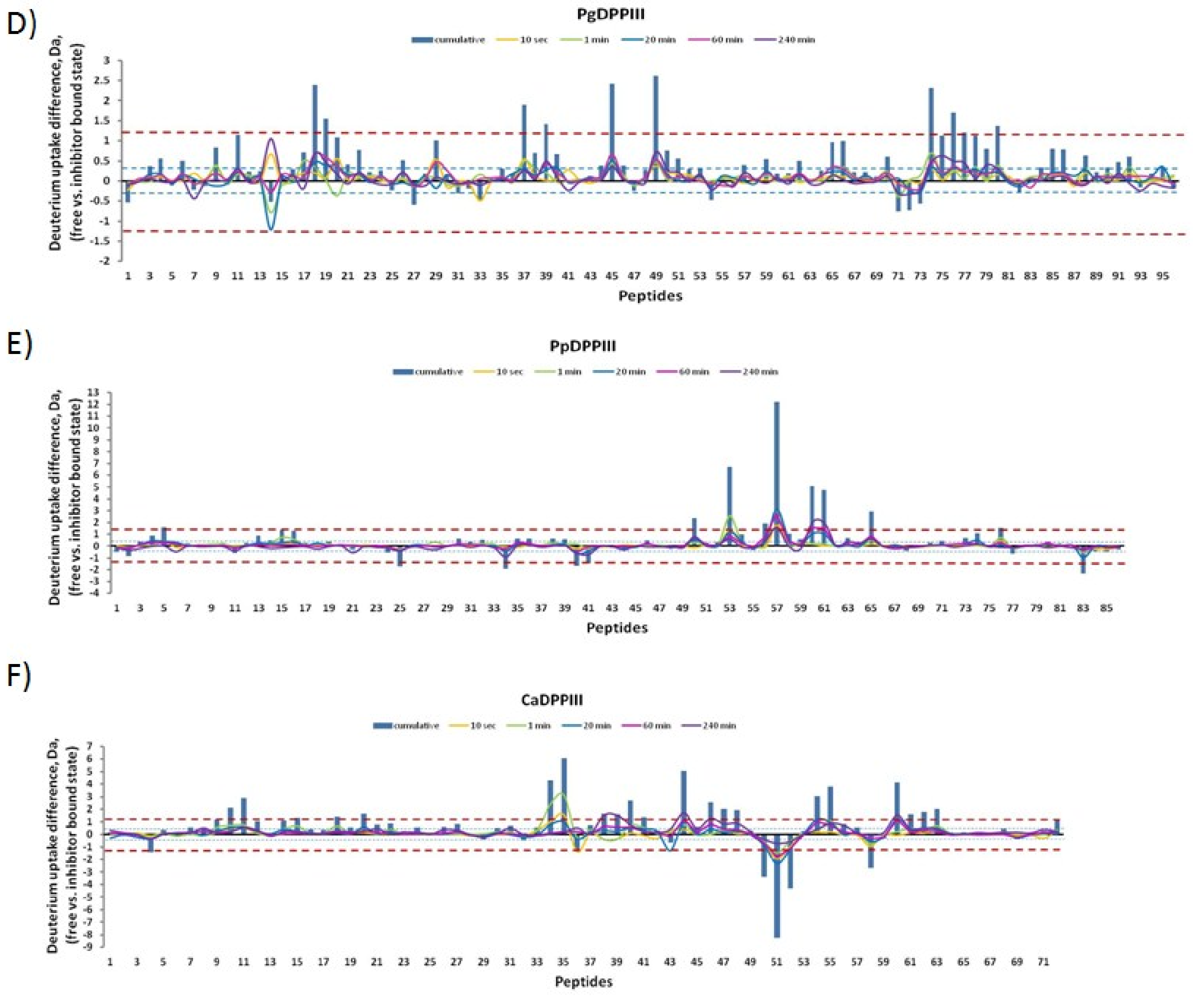

Figure S6. Graphical presentation of deuterium uptake differences for all peptides monitored in H/D experiments for the enzymes in unliganded and tynorphin complex states. Yellow, green, blue, pink and purple traces correspond to the 10 sec, 1, 10, 60, and 240 minute deuterium incubation time points, light blue dotted lines represent significance threshold limit of ±0.3 Da for each time point value. Difference cumulative value is sum of all time point values for each peptide and plotted as a vertical bar, dashed red lines denote significance threshold limit of ±1.3 Da for those values. A) H/D data difference plot for comparing unlinganded vs. tynorphin complex of human DPP III enzyme. B) H/D data difference plot for comparing unlinganded vs. tynorphin complex of DPP III enzyme from *Saccharomyces cerevisiae*. C) H/D data difference plot for comparing unlinganded vs. tynorphin complex of DPP III enzyme from *Bacteroides thetaiotaomicron*. D) H/D data difference plot for comparing unlinganded vs. tynorphin complex of DPP III enzyme from *Porphyromonas gingivalis*. E) H/D data difference plot for comparing unlinganded vs. tynorphin complex of DPPIII enzyme from *Physcomitrella patens*. F) H/D data difference plot for comparing unlinganded vs. tynorphin complex of DPP III enzyme from *Caldithrix abyssi*. For the labeled peptides identification (sequences) see Table S1.

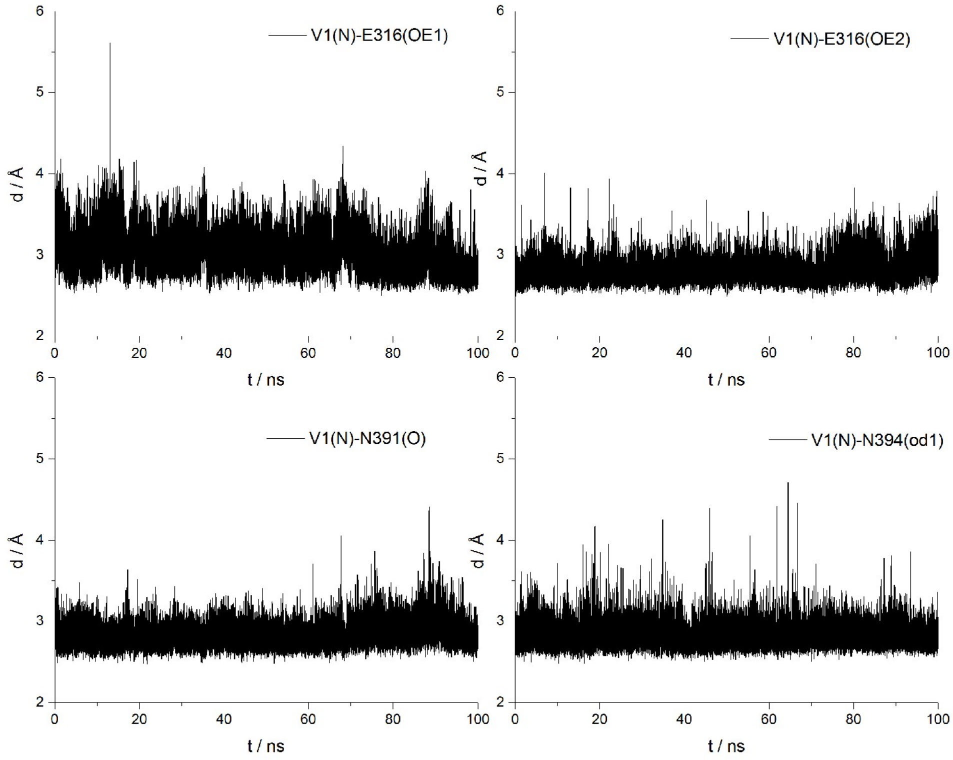
V1(N)-E316(OE1)
V1(N)-E316(OE2)
V1(N)-N391(O)
V1(N)-N394(od1)
d / Å
t / ns

a)

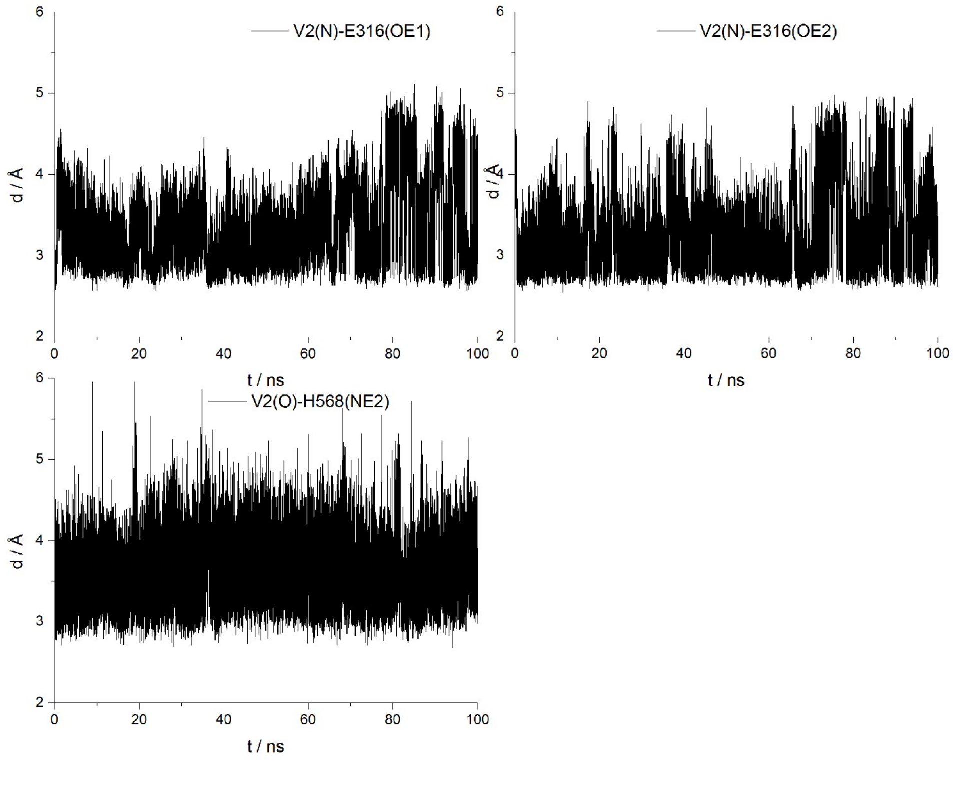
V2(N)-E316(OE1)
V2(N)-E316(OE2)
V2(O)-H568(NE2)
d / Å
t / ns

b)

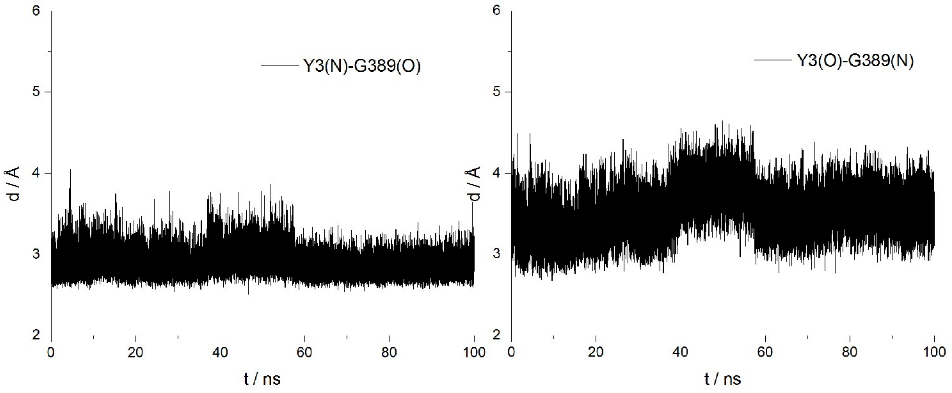

c)

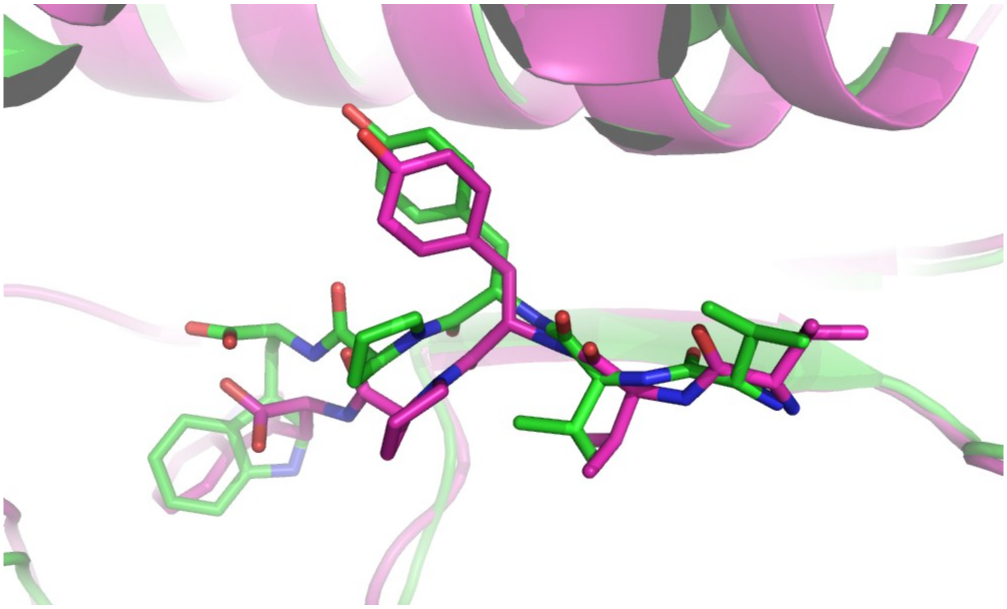

Figure S7. Top - changes of selected distances between substrate-protein hydrogen bond donor and acceptor atoms during MD simulations. Corresponding distances are show in Fig.5 by black dashed lines.

Bottom the overlay of tynorphin in the structures of the DPP III - tynorphin complexes obtained by MD simulation (initial – violet and final – the atom coded colors).

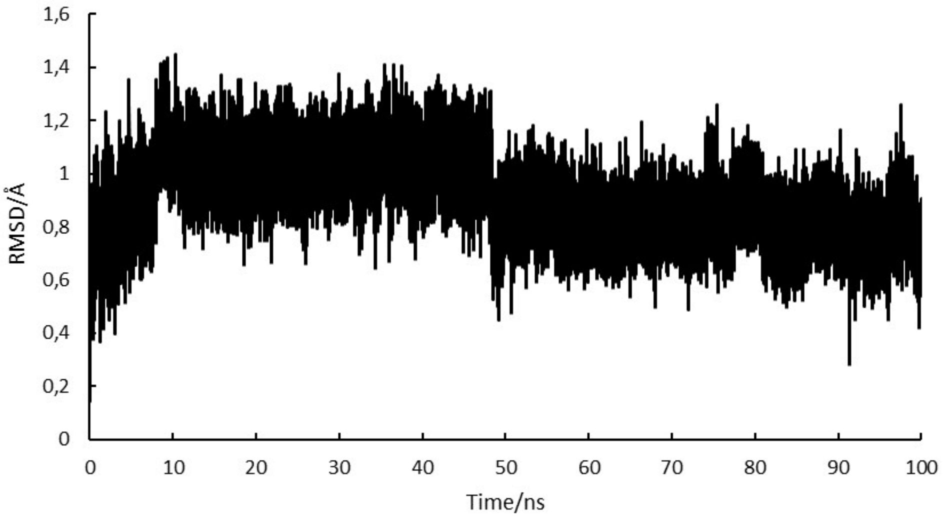
RMSD/Å
Time/ns

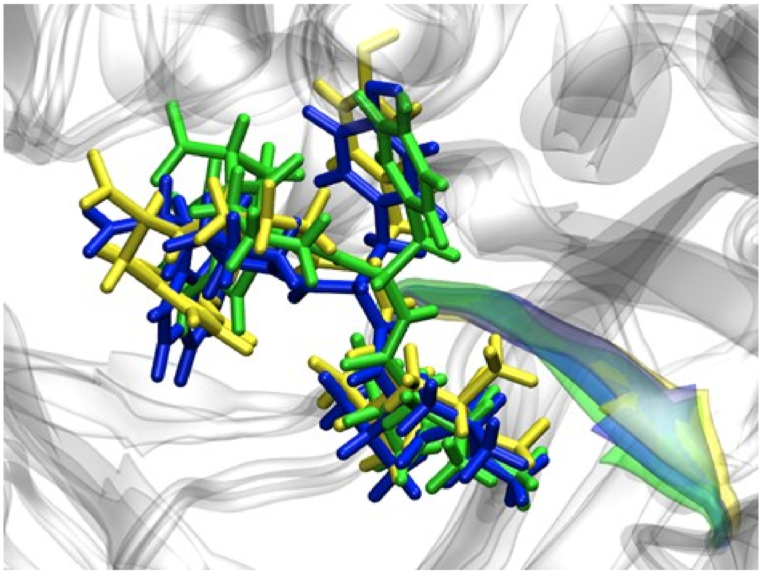

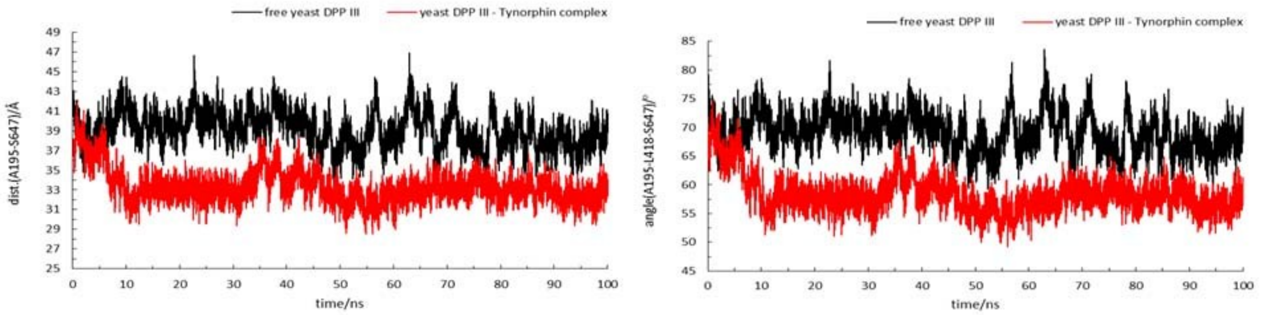
free yeast DPP III
yeast DPP III - Tynorphin complex
dist.(A195-S647)/Å
time/ns
angle(A195-L418-S647)/°

Figure S8 Top - the RMSD profile for the tynorphin backbone atoms during MD simulations of the yeast DPP III - tynorphin complex.

Middle – the overlay of the tynorphin in the yeast DPP III - tynorphin complexes sampled at 5ns (yellow), 50ns (blue) and 100ns (green) of MD simulations. Position of the ligand relative to the β-strand is given.

Bottom – Enzyme conformational change during 100 ns of MD simulation of free yeast DPP III and its complex with tynorphin, described by:(left) changes of distance between alpha carbons of residues from the "upper" and "lower" domain at the edge of the inter-domain cleft (Ser647 and Ala195, respectively) and (right) angle between residues at the inter-domain cleft edge and hinge (Ser647, Ala195 and Leu418, respectively).

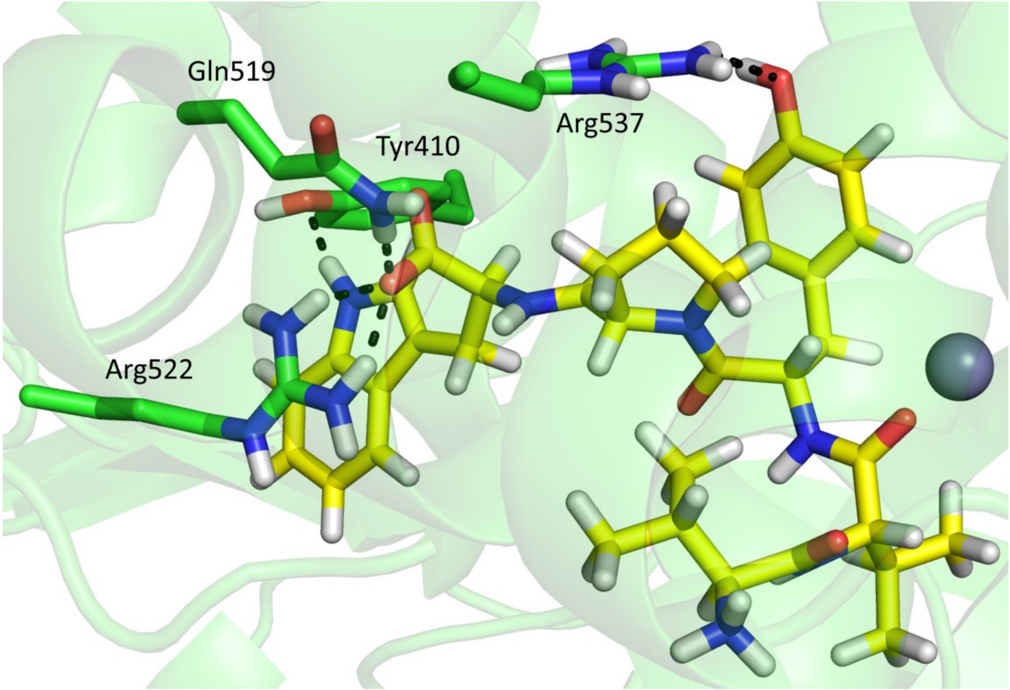

Figure S9. Tynorphin bound into the *Bt*DPP III active site as determined by molecular modeling (docking combined with MD simulations). Tynorphin and the selected amino acid residues from the enzyme active site are given in stick representation (coloured yellow and green, respectively) and the rest of the enzyme is shown as ribbon.

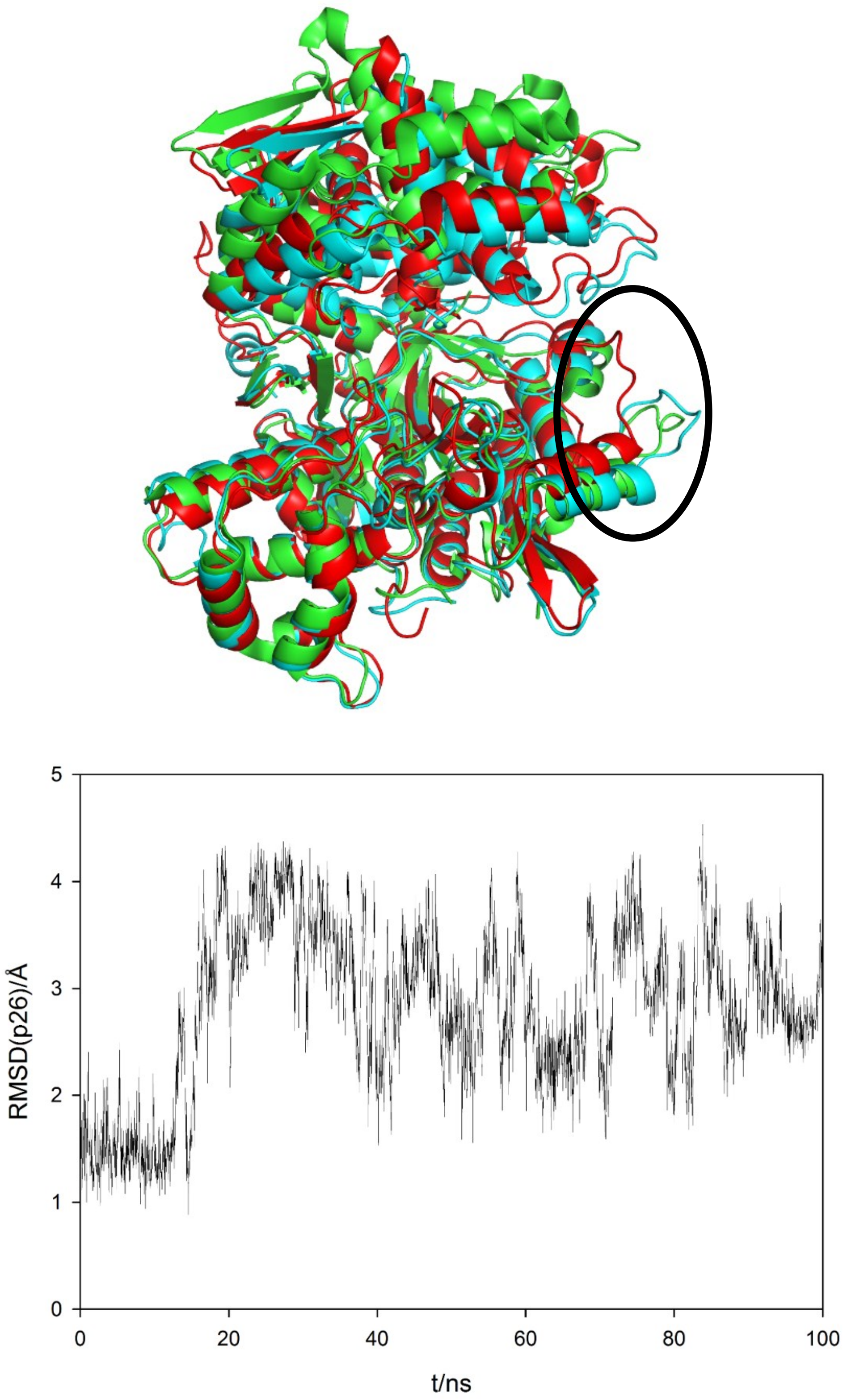

Figure S10. TOP - Alignment of the *Bt*DPP III structures, experimental, the open one (PDB code 5NA7; green), and those obtained by MD simulations of the ligand free protein (cyan) and its complex with tynorphin obtained after 100 ns of MD simulations (red). Loop described by peptide 26 is shown in black

ellipse. Ligand-free protein structure obtained by MD simulations (cyan) corresponds to the most closed MD structure, achieved with aMD calculations using the ff14SB force field. We used this particular structure to illustrate that p26 loop never, regardless of the degree of the protein (*Bt*DPP III) closure, undergoes translocation noticed in the complex with tynorphin.

BOTTOM - RMSD of the loop during MD simulation of the *Bt*DPP III – tynorphin complex. The plot clearly indicates that loop changed its position after about 20 ns of the simulation.

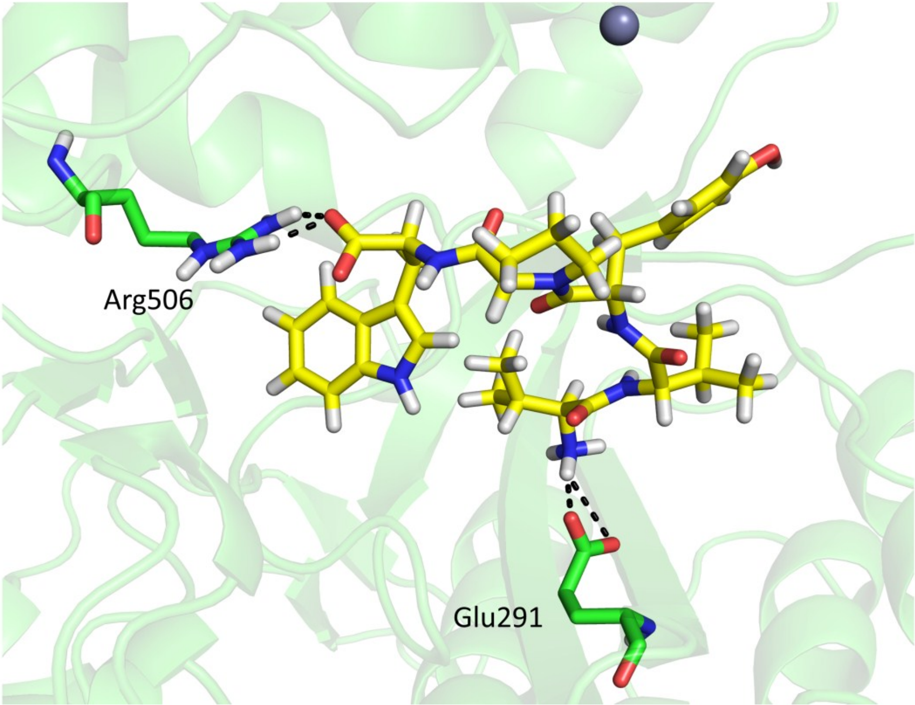

Figure S11. Tynorphin bound into the *Pg*DPP III active site as determined by molecular modeling (docking combined with MD simulations). Tynorphin and the selected amino acid residues from the enzyme active site are given in stick representation (coloured yellow and green, respectively) and the rest of the enzyme is shown as ribbon.

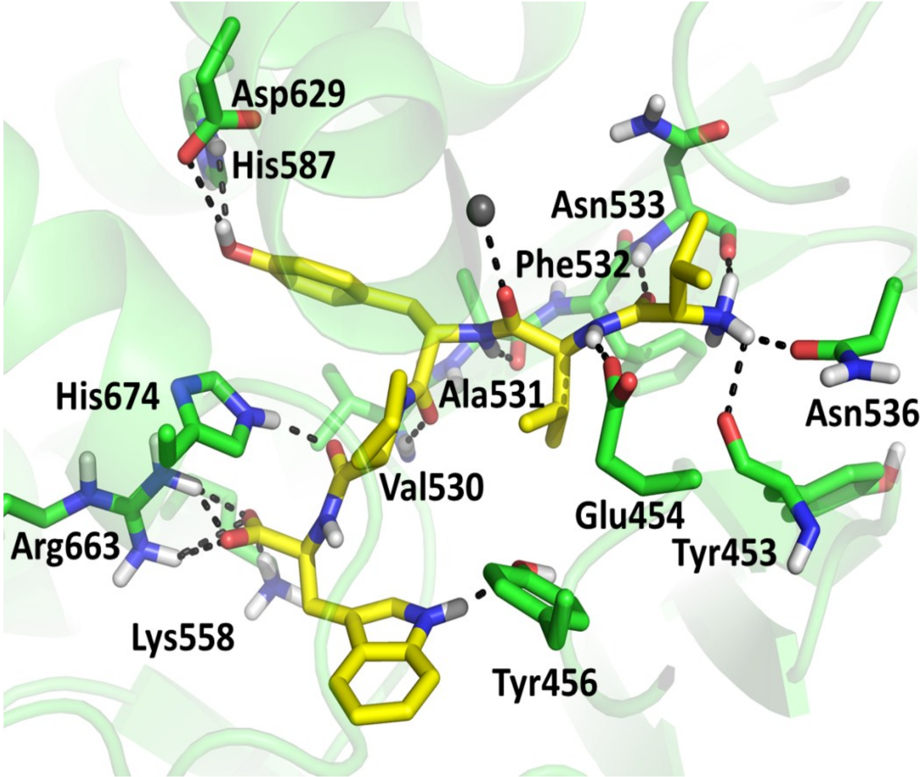

Figure S12. Tynorphin bound into the *Pp*DPP III active site as determined by molecular modeling. Tynorphin and the selected amino acid residues from the enzyme active site are given in stick representation (colored yellow and green, respectively) and the rest of the enzyme is shown as ribbon.

Table S1. Peptic peptides from all six DPP III orthologs analyzed for H/D exchange kinetics. Peptide numbers are related to their sequences and position in the primary structure of the enzyme.

No	Range	hDPP III peptides	No	Range	yDPP III peptides	No	Range	BtDPP III peptides	No	Range	PgDPP III peptides	No	Range	PpDPP III peptides	No	Range	CaDPP III peptides
1	6-17	YILPNDIGVSSL	1	4-14	FFADHDAPLSM	1	18-24	TAEADKF	1	11-17	GERIARF	1	1-7	MEGFQEE	1	30-39	VKLKRMIAQF
2	18-32	DCREAFRLLSPTERL	2	15-21	LSVKTEY	2	25-30	DYTVEQ	2	18-23	ADIEVL	2	8-14	FSLGECL	2	38-43	QFAPTE
3	33-40	YAYHLSRA	3	15-22	LSVKTEYF	3	25-31	DYTVEQF	3	30-39	FGTLTPKQRM	3	15-22	DVLTAAGL	3	45-51	KYDHSLL
4	41-46	AWYGGL	4	23-35	PQLTDKEQKYAHF	4	35-44	QILRYKVPEF	4	30-41	FGTLTPKQRMLC	4	55-63	LVLQKRADC	4	52-62	DERKQKVVENL
5	50-62	LQTSPEAPYIYAL	5	36-48	MSKASHAGSRVVM	5	45-55	ETLTLKQKELV	5	42-46	YHLSE	5	58-70	QKRADCKDSWPGL	5	63-70	YRAAKIMD
6	67-75	FRAQDPDQL	6	47-60	VMRQVSHESEPIFD	6	56-60	YYLTQ	6	49-63	LRGRDITTIQNCRYN	6	71-87	WDISSAGHISAGDTSLL	6	63-73	YRAAKIMDEIF
7	76-88	RQHALAEGLTEEE	7	64-76	AIHSKLNGKYPED	7	57-63	YLTQAAL	7	54-64	ITTIQNCRYNL	7	90-97	RRELQEEL	7	74-81	LDQVYSKN
8	89-93	YQAFL	8	90-94	YVSQF	8	64-69	EGRDIL	8	65-70	WVRSLM	8	98-105	GITLPADA	8	82-88	FEIREQL
9	94-100	VYAAGVY	9	95-101	LSNLGNF	9	70-78	FDQNGKYNL	9	71-85	ERIYTHLSKSERTDD	9	99-107	ITLPADAFE	9	89-97	RASSDPLDQ
10	101-106	SNMGNY	10	102-113	KSFGDTKFIPRC	10	79-86	RIRRMLEA	10	89-93	LEEYL	10	110-115	FDYLQE	10	89-98	RASSDPLDQL
11	107-123	KSFGDTKFVPNLPKEKL	11	111-117	PRCEVKF	11	87-100	VYTNYKGDKSAPDF	11	98-111	FANGIHHHYSGAKF	11	116-127	CVINGGKYINNE	11	89-100	RASSDPLDQLRL
12	124-131	ERVILGSE	12	118-124	FKQLLEL	12	101-105	KNMEV	12	112-119	IARFSPGF	12	128-133	YNDVYL	12	98-103	LRLEYF
13	132-143	AAQQHPEEVRGL	13	125-136	AKINPCSSPLTL	13	105-123	VYLKRVWFSNGIHHHYGME	13	120-124	LRAAL	13	134-144	VTLVDHIPVEA	13	103-113	FTIMFGPFDRL
14	144-150	WQTCGEL	14	137-150	SPVDVNHEFTSHHL	14	124-133	KFVPGFSQDF	14	125-138	REAGVELEPEEQVL	14	145-149	FTLQE	14	107-131	FGPFDRLNHDKPFIGNTPKPKGANF
15	151-155	MFSLE	15	151-157	FSTINEL	15	133-139	FLKQAVL	15	129-135	VELEPEE	15	146-153	TLQETEVS	15	131-139	FYPPDMTRE
16	152-170	FSLEPRLRHLGLGKEGITT	16	158-163	IDIGIY	16	140-145	GTDAQL	16	139-148	LERVLYDTDF	16	148-154	QETEVSA	16	137-143	TREEFEN
17	164-170	GKEGITT	17	160-171	IGIYHVEEKAAL	17	146-157	LPLSEGQTAEQL	17	150-161	PKQTEQSGEEDI	17	158-165	MKWQDYED	17	140-144	EFENW
18	171-178	YFSGNCTM	18	164-171	HVEEKAAL	18	158-162	CDELF	18	161-169	IIKASSVNF	18	166-186	AVRKEDSSYVPCDVDGGYSAL	18	145-154	LKAHPEDEAA
19	179-186	EDAKLAQD	19	172-178	LGFPSQG	19	163-171	PVMFDPAIL	19	163-169	KASSVNF	19	191-202	RRRYEVDTSAKL	19	155-159	FTSEF
20	184-188	AQDFL	20	172-182	LGFPSQGYTSA	20	172-182	AKRVNQADGED	20	172-186	PGITRAEAESHYKNL	20	203-207	EELQM	20	159-169	FTVIRRQDGKL
21	189-193	DSQNL	21	183-187	YYLGL	21	183-189	LVLTSAC	21	187-194	IEALPENE	21	208-229	HINRYVPVEIHGETTGLSKGDL	21	170-176	VAIPYSE
22	193-199	LSAYNTR	22	188-194	PVTPEDM	22	184-190	VLTSACN	22	195-201	KSCPPSF	22	230-238	LALSDTIRA	22	177-187	YYKEYLTRAAD
23	201-216	FKEVDGEGKPYYEVRL	23	197-202	LKEQLF	23	191-198	YYDGVTQQ	23	206-220	RLIRSTSGELKDEVC	23	234-241	DTIRAAKL	23	188-194	YLKKAAE
24	217-226	ASVLGSEPSL	24	205-213	LAILPENTR	24	199-203	EAESF	24	221-231	CIDGLYGPAIE	24	242-246	LDRIF	24	195-201	FADNPSL
25	227-236	DSEVTSKLKS	25	209-222	PENTRINKVGENSF	25	204-221	YGAMKDPKDETPVSYGLN	25	232-238	AVVASLE	25	247-251	LEQEW	25	202-207	KKYLQL
26	237-255	YEFRGSPFQVTRGDYAPIL	26	223-229	QIWVASE	26	225-241	VKEDGKIQEKVWKVGGL	26	238-252	EAAIPYTENEEQAAC	26	251-257	WATNPAL	26	208-213	RAEAFL
27	256-262	QKVVEQL	27	230-243	NVKNQITETYPSGQ	27	246-251	IEKIVY	27	253-258	IRLLCD	27	251-261	WATNPALKDSL	27	212-216	FLNND
28	263-280	EKAKAYAANSHQGQMLAQ	28	244-249	ITLSNA	28	252-272	WLKKAETVAENDAQKAVISKL	28	260-267	YRTGDIRL	28	262-270	QAASGKSEL	28	217-221	YYESD
29	281-299	YIESFTQGSIEAHKRGSRF	29	250-255	VTKVEF	29	259-272	VAENDAQKAVISKL	29	272-285	CIRWVENNRTRIDF	29	271-278	DRLKFAYY	29	222-227	LAWMDL
30	300-309	WIQDKGPIVE	30	256-266	IFGDHSREMRL	30	276-286	YETGSLKDFDE	30	286-292	INGFTEV	30	279-288	LINKSPWSIL	30	226-233	DLNDHTIE
31	310-314	SYIGF	31	267-271	VASYL	31	291-297	WVKDLDS	31	293-303	YADPIGIHGSW	31	289-295	DENDAFL	31	234-246	VVIGPYEVYEDKL
32	312-317	IGFIES	32	270-287	YLKEAQKFAANDTQKAML	32	298-304	RIDFVNG	32	304-309	EGLVHM	32	296-303	TTADSAIQ	32	247-253	FNYKAAF
33	318-328	YRDPFGSRGEF	33	290-308	YINHFVTGSSQAHKEAQKL	33	306-319	TESYGDPLGVKASW	33	312-323	EEAGRRTRIISE	33	304-311	IAEGAVDE	33	257-264	ITLRDPVE
34	331-343	FVAVVNKAMSAKF	34	309-323	WVKDISPVIETNIGF	34	309-319	YGDPLGVKASW	34	324-335	HAGWFEAHSPID	34	312-325	SVTWGGKVRYRAAF	34	265-274	SAKLKKFVGY
35	344-353	ERLVASAEQL	35	319-323	TNIGF	35	320-325	ESLVNF	35	328-338	FEAHSPIDARF	35	326-335	PVEKAPGANF	35	265-277	SAKLKKFVGYLDE
36	353-363	LLKELPWPPTF	36	327-336	YREPSGIIGE	36	326-339	KDLDATHRTEIISS	36	348-353	ATVVNV	36	336-340	YPVDM	36	278-300	MEKNLPIPDAYKNFNRGSESPMV
37	364-373	EKDKFLTPDF	37	341-352	VAIQNKERTAKF	37	337-343	ISSNAQW	37	354-367	LTIAGDSYPATPIG	37	341-345	DKEEF	37	302-306	VQEVF
38	379-398	LTFAGSGIPAGINIPNYDDL	38	353-362	SSLVNNAEEF	38	344-363	FEDHSPVDKSFKKEKVKGVS	38	368-374	INLPNAD	38	346-358	QAWKDGLDKGQQA	38	307-318	SAGDTKAGVQTL
39	392-398	IPNYDDL	39	362-371	FISLLPWSKD	39	364-369	AKVITA	39	375-386	WIRAEHGSKSVT	39	359-363	DATSF	39	307-319	SAGDTKAGVQTLA
40	399-413	RQTEGFKNVSLGNVL	40	372-382	YEKPIFNPPDF	40	373-381	AGDLYPATA	40	387-393	IDNITDA	40	364-383	FTTIRRSGDIHDADGAKVHD	40	318-326	LAFNLPNDE
41	414-426	AVAYATQREKLTF	41	387-406	VLTFTGSGIPAGINIPNYDD	41	382-390	IGINLPNAN	41	394-403	YNHAARGTGL	41	364-392	FTTIRRSGDIHDADGAKVHDLKIVPYSKE	41	319-326	AFNLPNDE
42	427-434	LEEDDKDL	42	407-422	VRLKIGFKNVSLGNIL	42	391-402	WIRAHHGSKSVT	42	404-412	YEEFIPDEE	42	393-397	YATIL	42	320-326	FNLPNDE
43	435-443	YILWKGPSF	43	419-424	GNILSA	43	403-409	IGNITDA	43	413-419	VRRHVEL	43	398-403	KEASYL	43	327-344	RVREAKGSKKVMLKNIHE
44	436-443	ILWKGPSF	44	425-436	AAKSSSKHPPSF	44	410-419	YNKAAHGNGF	44	420-427	HADLTDSL	44	404-416	LHKAGDSAENPSL	44	345-356	AKFDKLLKPIAE
45	444-449	DVQVGL	45	437-446	ISQEDRPIFE	45	423-427	FVCND	45	432-457	HECLGHGSGQLLPGVPGDALGEHAST	45	417-426	KRLLKAKADA	45	351-356	LKPIAE
46	450-460	HELLGHGSGKL	46	447-453	KYQSDSF	46	428-443	EERQRIDQYGDLTGEL	46	458-465	LEETRADL	46	427-431	FLSND	46	352-359	KPIAEKVL
47	461-469	FVQDEKGAF	47	453-470	FEVQVGIHELLGHGSGKL	47	448-463	HECLGHGSGKLLPGVD	47	466-470	FALYF	47	432-436	YYESD	47	360-369	FAEQLPLVTF
48	470-474	NFDQE	48	463-470	LGHGSGKL	48	464-473	PDALKAYGST	48	470-476	FLADPKM	48	437-442	IAWMEL	48	361-368	AEQLPLVT
49	475-484	TVINPETGEQ	49	471-479	LTEFTDGFN	49	474-481	IEEARADL	49	479-487	LGLLTDPDA	49	440-447	MELDSPLD	49	369-378	FEGFFNHTLM
50	484-488	QIQSW	50	480-489	FDKENPPLGL	50	485-492	YYVADPKL	50	482-492	LTDPDAYKANY	50	448-457	VTIGPYETYE	50	379-392	HEISHGLGPGKIVL
51	489-499	YRSGETWDSKF	51	490-497	DGKPVSTY	51	493-503	VELKLVPDAEA	51	493-501	YKYMLNGLM	51	456-461	YEDGLF	51	393-412	NGRQTEVKKELKETYSSIEE
52	500-506	STIASSY	52	498-511	YKVGETWGSKFGQL	52	504-508	YKAEY	52	513-523	IEEAHMRNRAL	52	461-467	FGYKATF	52	399-412	VKKELKETYSSIEE
53	510-516	RAESVGL	53	512-518	AGPFEEC	53	511-516	FLMNGL	53	524-530	IARYVLE	53	471-484	IGIRDDEATQRLKL	53	413-420	CKADVLGM
54	517-525	YLCLHPQVL	54	519-525	RAEVIAM	54	516-520	LMTQL	54	531-540	HAERPGAMSL	54	485-491	FSHNLQE	54	421-425	YNNLF
55	526-534	EIFGFEGAD	55	526-539	FLLTNKKILDIFGF	55	520-528	LVRIEPGNN	55	541-549	VCEEGKTAL	55	492-501	MEDNLPMDDE	55	425-441	FMIEKGVYPPEFEKQIY
56	526-537	EIFGFEGADAED	56	540-550	HDVESQDKVIY	56	529-541	IEEAHMRNRQLIA	56	550-555	VIKDYE	56	502-517	YKSKTVTFSPIRVIQL	56	440-445	IYVTFL
57	538-543	VIYVNW	57	551-556	AGYLQM	57	540-545	IARWVF	57	556-564	AVRAIIAGL	57	518-532	LYNSGDVKGPQTVAF	57	443-452	TFLAGIFRTI
58	541-546	VNWLNM	58	557-563	ARAGLLA	58	546-555	EKGAPDKVVE	58	565-576	LTEVQRIKSEGD	58	532-538	FNLPNDD	58	450-457	RTIRFGIN
59	547-551	VRAGL	59	562-583	LALEYWNPKTGKWGQPHMQARF	59	556-569	MVKKDGKTYVVVND	59	568-583	VQRIKSEGDYTAGKAL	59	539-548	RIVKERGTAM	59	458-468	EAHGAGNAVIF
60	552-556	LALEF	60	587-598	KTFMKHSTDKNF	60	570-580	YEKVRQLFGEL	60	583-589	LVERYAV	60	549-559	VMLKNISQAKF	60	472-477	LEKGAY
61	557-562	YTPEAF	61	599-603	LKLEM	61	581-593	LAEIQRIKSTGDF	61	588-597	AVHVDPLLHE	61	551-559	LKNISQAKF	61	472-483	LEKGAYQFDPAA
62	563-569	NWRQAHM	62	604-610	NSTNDDF	62	584-593	IQRIKSTGDF	62	598-606	EVLTRYAKL	62	560-564	DYILL	62	481-490	PAAHRVKVNF
63	577-585	RVLLEAGEG	63	611-618	AIKLDKSL	63	599-603	LVENY	63	607-613	DIAPYKG	63	564-568	LPIAN	63	491-505	EKIKDGVRDLANKVL
64	586-601	LVTITPTTGSDGRPDA	64	615-629	DKSLIKTAGHECVKD	64	604-611	AVKVDPAL	64	614-623	FVNPRLRPVY	64	565-570	PIANVC	64	506-513	TIQAQGDY
65	606-618	DRSKIRSVGKPAL	65	630-641	YLKHLHVYKCSG	65	617-629	ARYKKLNLAPYKG	65	614-631	FVNPRLRPVYNSEGRLTD	65	571-581	VEASQRGAVDF	65	514-519	MAAKNL
66	622-628	LRRLQVL	66	642-649	DVEQGSKY	66	630-636	FINPVYE	66	607-635	DIAPYKGFVNPRLRPVYNSEGRLTDATIE	66	585-592	FTHTICHE	66	515-522	AAKNLLET
67	622-639	LRRLQVLKSTGDVAGGRA	67	650-657	FIDRSTVT	67	637-647	LVTDKDGNITD	67	634-639	IEYTEG	67	593-611	CCHGIGPHNIVTPDGRAST	67	523-528	YAVESE
68	629-639	KSTGDVAGGRA	68	655-660	TVTPDL	68	648-654	VTVSYNE	68	640-644	YAEQM	68	612-616	VRLEL	68	524-531	AVESEPIM
69	640-645	LYEGYA	69	666-680	IVLSKRLPRRQFIQS	69	656-661	YVEQML	69	644-650	MLRYSAE	69	617-622	QEVYSA	69	532-537	IMRARL
70	645-655	ATVTDAPPECF	70	681-688	NSYIDDNN	70	662-683	RYSKDYSPLPSVNNLEHHHHHH	70	651-663	YSFLPTDSPLLQE	70	623-633	IEEAKADIVGL	70	538-543	QELPVD
71	656-660	LTLRD	71	685-694	DDNNKVTLKE				71	664-679	ARRLRSHLRRAMDGVL	71	634-638	WALHF	71	541-549	PVDIKPIFQ
72	664-679	LRKESRKLIVQPNTRL	72	695-703	YDETPQGML				72	680-693	SASMREKGLHYGIN	72	639-650	LVDKGLLPRSLE	72	550-563	IEKELGNSNLEHHH
73	684-688	VQLLE							73	694-701	FGVTREHL	73	639-653	LVDKGLLPRSLENTM			
74	689-696	YEASAAGL							74	702-716	LRLARTADASAPLAD	74	653-657	MYVSF			
75	697-711	IRSFSERFPEDGPEL							75	702-718	LRLARTADASAPLADYL	75	658-662	LAGCF			
76	714-718	ILTQL							76	705-716	ARTADASAPLAD	76	662-681	FRSIRFGLEEAHGKGQALQF			
77	719-725	ATADARF							77	705-717	ARTADASAPLADY	77	682-686	NFILE			
									78	719-731	WRRDVRETKILAT	78	685-690	LEKGGF			
									79	726-731	TKILAT	79	691-698	SYHPDGTF			
									80	731-738	TMIFPAEE	80	699-711	SVDYTKVRQCVED			
									81	732-738	MIFPAEE	81	711-717	DLSRLIL			
									82	732-739	MIFPAEEL	82	718-731	TIEAKGDKAGAEAL			
									83	738-747	ELTHEQATRL	83	732-746	LAKYAALTPQLQQSF			
									84	748-755	LREADNVE	84	747-759	DALQDVQVPVDIF			
									85	756-764	LREQLTANL	85	760-766	PTFKLLD			
									86	765-771	LERMPEA	86	763-767	KLLDD			
									87	772-778	IRSIGRW						
									88	779-788	IESKETTPDM						
									89	789-795	MTGVLTL						
									90	800-814	FTRGIFPEDVPAEKL						
									91	817-822	LAILHL						
									92	821-831	HLSDEEQKAEL						
									93	855-868	CLLPESSQDTAPVL						
									94	869-873	YELCE						
									95	874-879	DIRFEL						
									96	878-894	ELDFYPKDELEHHHHHH						

Table S2. Percent of amino acid identity matrix

hDPPIII	100	36.4	23.95	23.65	19.52	22.72
yDPPIII	36.4	100	21.77	22.69	17.41	19.33
*Bt*DPPIII	23.95	21.77	100	49.92	22.89	22.31
*Pg*DPPIII	23.65	22.69	49.92	100	20.34	22.09
*Pp*DPPIII	19.52	17.41	22.89	20.34	100	42.21
*Ca*DPPIII	22.72	19.33	22.31	22.09	42.21	100

Table S3. Mean number of amide H-bonds per residue in selected peptides in different hDPP III structures. Peptides with higher deuterium uptake in complex than in ligand free enzyme are given in red, and for those with lower deuterium uptake in complex than in free enzyme are given in blue.

Peptide	Open hDPP III	Closed hDPP III	Closed hDPP III – tynorphin
Pep2 [18-32]	0.533	0.333	0.933
Pep13 [132-143]	1.000	1.000	0.667
Pep28 [263-280]	0.611	0.889	0.556
Pep29 [281-299]	1.368	1.105	1.474
Pep34 [331-343]	1.154	0.769	1.077
Pep38 [379-398]	0.600	0.800	1.050
Pep39 [392-398]	0.571	0.571	1.000
Pep40 [399-413]	0.867	0.933	1.000
Pep55 [526-534]	0.778	0.333	0.333
Pep56 [526-537]	0.917	0.333	0.333
Pep62 [563-569]	0.571	0.857	1.000
Pep64 [586-601]	0.750	0.750	0.813
Pep75 [697-711]	0.800	0.600	1.067

Table S4. Amino acid residues from DPP III orthologs interacting with tynorphin residues electrostatically during MD simulations. In some cases these interactions satisfy criteria for hydrogen bond interaction. The residues for which these criteria were satisfied for at least 1% of the simulation time are outlined. The amino acid residues that belong to peptides for which changes in deuterium uptake upon tynorphin binding were determined are represented by bold letters.

Complex	S_2 (NH3-Val)	S_1 (Val)	S_1' (Tyr)	S_2' (Pro)	S_3' (Trp-COOH)
hDPP III	Glu316[1] *94%*	Glu316[1] *67 %*	**Pro387**	Phe109	**Ile386** *26 %*
	Tyr318[1]	Tyr318[1]	**Ala388**[2] *83%*	**Pro387**	**Pro387** *5%*
	**Gly389**[2]	**Pro387**	**Gly389**[2] *68%*	**Ala388**[2]	**Ala388**[2]
	**Ile390**[2]	**Gly389**[2]	**Ile390**[2]	Phe443[3]	**Val412**[6] *22%*

	**Asn391**[2] *44%*	**Ile390**[2]	Phe443[3]	**Gln566**	Ala416
	**Ile392**[2]	Ser442[3]	Gln446[3]	**His568**[5,8]	Phe443[3]
	**Asn394** *81%*	Glu508	Val447[3]	Arg572 *14 %*	Arg669[7] *125%*
	**Arg399**[9]	**His568**[5,8] *2%*	His450[4]	Arg669	Lys670[7]
	His455[4]	Met547	Glu508[4]		Ile672
	Glu508		Glu512[4] *89%*		
			**His568**[5,8]		
			Arg572 *1.8%*		
yDPP III	Ile324	Phe104	Phe104	**Gln576**[5] *2%*	**Gly394**[2] *6%*
	Glu325[1] *4%*	Glu325[1] *1%*	**Pro396**	**His578**[5]	**Ile395** *35%*
	**Tyr327**[1] *1%*	**Tyr327**[1]	**Ala397**[2]	**Met579**[5]	**Pro396**
	**Gly398**[2]	**Gly394**[2]	**Gly398**[2] *81%*	Arg674[7]	**Ala397**[2]
	**Ile399**[2]	**Gly398**[2]	Leu422		Ile421[6]
	Asn400[2] *100%*	Asn400[2]	Phe453[3]		**Ala425**
	**Ile401**[2] *2%*	**Ile401**[2]	Val457[3]		**Tyr566**
	**Asn403** *17%*	**Gln576**[5]	**Gln576**[5]		Arg674[7] *90%*
	**Arg408**[9]		**His578**[5] *6%*		Arg675[7]
	**Gln576**[5]		Arg674		Phe677
*Pg*DPP III	Glu299 *44%*	Thr290[1]	**Ser456** *1%*	Glu514[5] *1%*	His105 *1%*
	Glu304 *26%*	Glu299 *26%*	Gu515[5] 11%	Gu515[5]	Tyr106 *7%*
	**Asp359** *44%*	Val292[1] *1%*	Ala516[5]	Ala516[5]	**Asp359**
	**Ser360** *27%*	Tyr293[1] *1%*			**Pro362**
	**Pro365** *1%*				**Ala363**
	**Gly367**[2]				Arg506[8] *57%*
					Lys508 *40%*
	Ile368[2]				Glu514[5] *2%*
	Asn369[2]				Glu515[5] *5%*
					Tyr69 *2%*
					Lys612
*Bt*DPP III	**Asn385**[2]	**Ile382**[2]	**Ile382**[2] *2%*	His448 *1%*	Tyr120
	Arg393[9] *1%*	**Gly383**[2] *5%*	**Gly383**[2]	Glu476[4]	Ala379
	Ser398	**Ile384**[2] *1%*	Thr402 *2%*	**His533**[5] *7%*	Thr380 *1%*
	Ser400 *3%*	**Asn385**[2] *1%*	Thr407	**Asn536** *20%*	Ala381 *2%*
	His453	Arg393	Tyr410	**Arg537** *5%*	**Ile382**[2] *3%*
	Ser472[9] *6%*	Ser400 *1%*	Gly441		Gly383[2]
	Glu476[4] *1%*	Thr402 *1%*	His444 *6%*		Tyr410 *7%*
	**Ala532** *1%*	**His533**[5] *5%*	Thr445 *24%*		Asn49 *1%*
	**His533**[5] *1%*		His448		Asn514 *1%*
			Glu449		Gln519 *14%*
			**His533**[5]		**Arg522** *87%*
			**Arg537** *34%*		**Ile523**
					**Glu531**[5]
					**His533**[5] *33%*
					**Met534**[5] *1%*
					**Arg537** *30%*
					Tyr627[7]

					Lys628[7] *1%*
					Phe630
*Ca*DPP III	Glu240[1] *97%*	Glu240[1] *69%*	**Thr317** *1%*	Tyr242[1] *2%*	Leu113 *5%*
	Tyr242[1] *5%*	Tyr242[1] *5%*	**Leu318** *70%*	**Val315**[2] *4%*	**Gly314** *2%*
	**Asn321**[2] *35%*	**Val315**[2] *1%*	**Ala319**[2] *43%*	**Thr317** *1%*	**Val315**[2] *21%*
	**Leu322**[2]	**Thr317** *5%*	His375 *9%*	**Leu318**	**Gln316** *21%*
	**Asn324**[2] *3%*	**Phe320** *3%*	Thr376	His460[5] *7%*	**Leu318**[2] *1%*
	Arg329[9]	**Asn321**[2] *6%*	**Glu380** *2%*		**Lys346**
	**His383**[4] *1%*		Asp416 *77%*		**Arg450** *149%*
	**Tyr407**		Phe444		**Thr451** *96%*
	**Lys400** *2%*		His460[5] *3%*		**Arg453** *2%*
	**Glu411**[4] *2%*				**Phe454** *2%*
					Glu458[5]
					His460[5] *91%*

[1]is related to the region highly conserved among the studied orthologs.

^{313}GFIESYRDP human
^{322}GFIETYREP yeast
^{304}GFTESYGDP *B. thetaiotaomicron*
^{288}GFTEVYADP *P. gingivalis*
^{237}GPYEVYEDK *C. abyssi*

[2]is related to the region highly conserved among the studied orthologs.

^{388}AGINIPN human
^{397}AGINIPN yeast
^{382} IGINLPN *B. thetaiotaomicron*
^{366}IGINLPN *P. gingivalis*
^{318}LAFNLPN *C. abyssi*

[3]conserved in human and yeast

[4]The conserved signatures HEL(C)LGH and EECR(K)AE(D). In CaDPP III instead of hexapeptide HEL(C)LGH there is pentapeptide HEISHG

[5] related to the conserved region:

^{564}WRQA**H**MQ human
^{574}WGQP**H**MQ yeast
^{529}IEEA**H**MR *B. thetaiotaomicron*
^{513}IEEA**H**MR *P. gingivalis*
^{456}IEEN**H**GA *C. abyssi*

It should be noted that **H** is conserved in all orthologs, while IEE(N)A**H** in the bacterial ones (*B. thetaiotaomicron*, *P. gingivalis* and in *C. abyssi* (INEAH).

[6] V in human and in other orthologs

[7]RK in human, RR in yeast and YK in *Bt* and *Pg*

[8]Conserved in the bacterial orthologs

[9]Conserved in all orthologs

Table S5. Number of hydrogen bonds per amino acid for the selected peptides in the open and closed ligand-free *Ca*DPP III structures as and its complex with tynorphin. Residues which experience the increase of deuterium uptake in the *Ca*DPP III – tynorphin complex are shown in bold face representation. The number in parentheses denotes number of amino acid residues in peptide.

Peptide	Open *Ca*DPP III	Closed *Ca*DPP III	Closed *Ca*DPP III – tynorphin complex
11 (12)	0.583	0.500	1.166
39 (13)	0.846	0.846	0.308
40 (9)	0.778	0.444	0.444
44 (12)	1.083	0.917	0.833
46 (8)	1.000	1.000	1.000
**50 (14)**	0.571	0.714	0.642
**51 (20)**	0.850	0.850	0.700
52 (14)	1.000	0.929	0.643
54 (5)	1.6	0.200	0.600
55 (17)	0.882	0.353	0.470
**58 (8)**	0.500	0.375	0.125
61 (6)	0.833	0.833	0.667
